# PSMG2 role in tumorigenesis and stemness mediated by protein accumulation, reticulum stress and autophagy

**DOI:** 10.7150/ijbs.105263

**Published:** 2025-03-21

**Authors:** Asunción Espinosa-Sánchez, Elena Blanco-Alcaina, Amancio Carnero

**Affiliations:** 1 Instituto de Biomedicina de Sevilla (IBIS)/HUVR/CSIC, Hospital Universitario Virgen del Rocío, Ed. IBIS, Consejo Superior de Investigaciones Científicas, Universidad de Sevilla, Avda. Manuel Siurot S/N, 41013, Seville, Spain.; 2 CIBER de Cancer (CIBERONC), Instituto de Salud Carlos III, Madrid, Spain.

**Keywords:** PSMG2, proteasome, tumorigenesis, dedifferentiation, ER stress.

## Abstract

The analysis of the dedifferentiation process has suggested that differentiated tumor cells undergo transformation toward cancer stem cells, accompanied by an increase in resistance to current chemotherapeutic treatments. Head and neck cancer (HNSCC) is a tumor with a high incidence and bad prognosis, and it is necessary to identify genes with alterations that can be explored therapeutically. PSMG2 is a chaperone protein that forms a heterodimer with PSMG1 and promotes the assembly of the 20S proteasome. Here, we characterized the effect of PSMG2 downregulation on tumorigenesis and the dedifferentiation process in head and neck cancer cell lines. We observed that high PSMG2 levels are associated with poor prognosis and survival in patients with HNSCC. Knockdown of PSMG2 reduced proliferation *in vitro* and *in vivo* in HNSCC cell lines. Moreover, the downregulation of PSMG2 diminished stemness, dedifferentiation and reprogramming properties. The reduction in PSMG2 levels caused the accumulation of polyubiquitinated proteins, increasing endoplasmic reticulum (ER) stress and activating apoptosis and autophagy as compensatory mechanisms. Furthermore, the response to proteasome inhibitors was increased in low-level PSMG2 patients. Therefore, PSMG2 is implicated in the assembly of the proteasome, which regulates ER stress as an essential cellular mechanism and autophagy and apoptosis as compensatory mechanisms for cellular homeostasis. PSMG2, and by extension the proteasome, is involved in cellular reprogramming and stemness.

## Introduction

The dedifferentiation process or cellular plasticity is the process in which differentiated or somatic cells are converted into undifferentiated cells of their own lineage and develop self-renewal and pluripotency properties 1, 2. Numerous theories have been formulated to explain the appearance of cancer 3-5. The hierarchical model of cancer is based on cancer stem cells (CSCs), which show pluripotency and self-renewal properties. CSCs remain in the tumor indefinitely by self-renewal and differentiate into the different cell subtypes that form the tumor bulk 6. CSCs are responsible for the initiation, development and appearance of tumor recurrence and/or metastasis 3. Identification of the cellular dedifferentiation process has suggested that differentiated tumor cells, through the direct or indirect activation of oncogenic transcription factors, undergo a transformation toward CSCs, acquiring self-renewal and pluripotency properties 7-10. These signs of dedifferentiation are accompanied by an increase in resistance to current chemotherapeutic treatments 11, 12 and, in some cases, to radiotherapy 13, 14. Another important characteristic of CSCs is their ability to switch to a dormant or inactive state. CSCs can enter the G0 phase of the cell cycle (quiescent) 15, and since most of the current cancer therapies require a high rate of proliferation, G0-arrested cancer stem cells can escape these treatments. CSCs would be able to recover their most proliferative state when conditions are favorable and regenerate the tumor 16-20. Consequently, CSCs represent a problem in the therapeutic approach, being one of the main causes of tumor relapses. In recent years, studies have focused on the search for surface markers and transcriptional signatures specific to the cancer stem cell phenotype and mechanisms to eliminate these cells from the body.

Head and neck cancer (HNSCC) is the seventh most common tumor with the highest incidence worldwide. Approximately 1,000,000 new cases of HNSCC are diagnosed each year and are often diagnosed in advanced stages 21. Head and neck cancer comprises a group of tumors in different anatomical locations, including the oral cavity, tongue, pharynx, nasopharynx, oropharynx, hypopharynx, larynx, and salivary glands. The most common head and neck cancers originate in the mucosal epithelium of the oral cavity, pharynx, and larynx. Generally, cancers of the oral cavity and larynx are associated with tobacco consumption and alcohol abuse 22-25. Pharyngeal tumors are currently attributed to human papillomavirus (HPV) infection 26. Head and neck cancers can have a relatively poor prognosis due to several factors. They are detected at late stages, making it more difficult to treat and reducing the chances of a favorable outcome. They have a tendency to spread locally, increasing the risk of recurrence and making surgical removal of the tumor more challenging. Individuals with head and neck cancers are at an increased risk of developing second primary tumors in the head and neck region or other areas of the body. The occurrence of multiple primary tumors can complicate treatment and reduce overall survival rates. Finally, the treatment options available are currently very limited, mostly involving surgery and radiation therapy, and limited chemotherapy options limit the survival of patients. Therefore, it is necessary to explore the biology and pathology of these tumors to identify vulnerabilities that can be explored therapeutically.

PSMG2 is a chaperone protein that forms a heterodimer with PSMG1. This heterodimer promotes assembly of the 20S proteasome and prevents the dimerization of the α ring 27. The assembly of the 20S proteasome (core particle) is mediated by at least 5 extrinsic chaperones, PSMG1-4 and UMP1/POMP, in mammals 27. The process begins with the formation of the alpha ring supported by the complex of the heterodimeric chaperones PSMG1-PSMG2 and PSMG3-PSMG4 28-32. When the α ring is complete, the β subunits are incorporated onto it. This process is mediated by propeptides, the C-terminal group of β subunits and assembly chaperones. The order of incorporation of the β subunits is as follows: β2, β3, β4, β5, β6, β1 and β7 33-35. β2 recruitment is dependent on the UMP1 chaperone 34, 36. The PSMG3-PSMG4 complex dissociates from the α ring when β3 is incorporated. The dimerization of the 20S proteasome occurs after the incorporation of the last subunit, β7, forming a mature 20S subunit. Finally, the propeptides of the β-subunit and the heterodimer PSMG1-PSMG2 are cleaved and degrade UMP1/POMP 28, 37. The proteasome is a multienzymatic complex that maintains cellular homeostasis by degrading nonfunctional, misfolded, damaged and/or foreign proteins. The ubiquitin‒proteasome system (UPS) is the major quality control system in eukaryotic cells and is responsible for approximately 80-90% of protein degradation. The 26S proteasome is composed of a 20S core catalytic unit and two 19S regulatory units. The 19S regulatory units are responsible for recognizing and cleaving the ubiquitin chains, unfolding the proteins to be degraded and sending them to the central cavity of the 20S catalytic subunit to be degraded. The proteasome plays an essential role in many basic cellular processes, such as cell cycle progression, signal transduction, cell death, immune responses, protein processing and quality control 27.

We described here that high PSMG2 levels are associated with poor prognosis and survival in HNSCC. Moreover, the response to proteasome inhibitors could be altered according to the level of PSMG2. To explore the role of PSMG2 in tumorigenesis and the dedifferentiation process, we performed functional assays with the knockdown of PSMG2 *in vitro* and *in vivo* in two head and neck cancer cell lines. We focused on the alteration of proteasome activity when the level of PSMG2 was reduced and the activation of various mechanisms to maintain cellular homeostasis, such as endoplasmic reticulum stress, apoptosis and autophagy.

## Results

### Differential PSMG2 expression and prognostic implications in cancer

**Figure 1:**
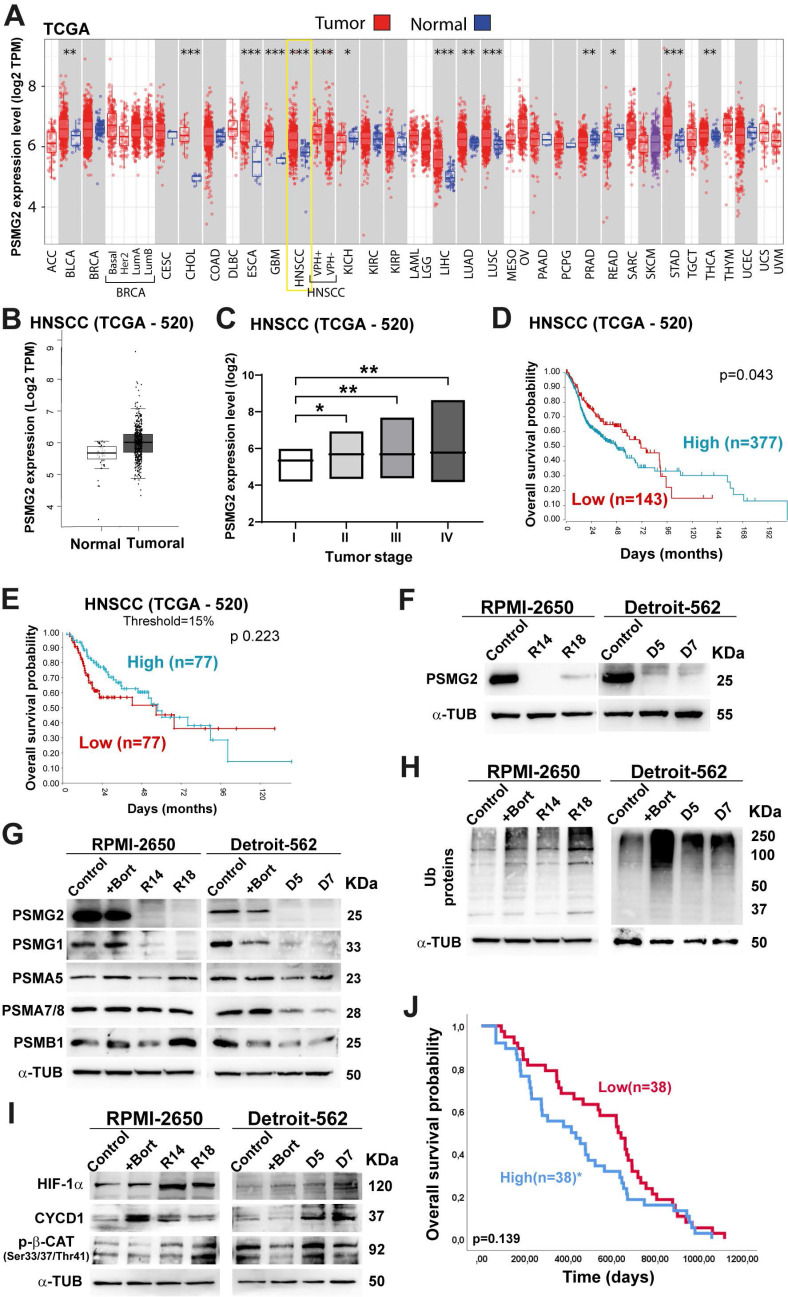
** Differential expression of PSMG2 in cancer and could act as a prognostic marker. (A)** Differential mRNA expression of PSMG2 in tumor and normal tissues in some type of cancers by TIMER 2.0 using TCGA database. The meaning of the acronyms is provided in File S1. The statistical analysis was computed by the Wilcoxon test (*: p-value < 0.05; **: p-value <0.01; ***: p-value <0.001). **(B)** Expression level of PSMG2 in non-tumoral and tumoral tissue in HNSCC by GEPIA using TCGA database. **(C)** Comparison of the expression of PSMG2 in the different tumor stage (I, II, III and IV) in HNSCC. **(D-E)** Overall survival of patients with high versus low expression of PSMG2. **(D)** Kaplan-Meier survival analysis was performed for the entire patient cohort (n=520). **(E)** Kaplan-Meier survival analysis comparing overall survival between the top 15% highest and bottom 15% lowest PSMG2 expression groups. **(F)** Validation of the reduction in PSMG2 expression in CRISPR PSMG2 cells of the RPMI-2650 and Detroit-562 head-neck cancer cell lines by Western blot analysis.** (G)** Measurement of the protein levels of PSMG2, PSMG1 and other alpha and beta subunits of the 20S proteasome in RPMI-2650 and Detroit-562 control cells, control cells treated with bortezomib (+bort) and CRISPR PSMG2 cells by western blot analysis. **(H)** Analysis of ubiquitinated protein levels in RPMI-2650 and Detroit-562 control cells, control cells treated with bortezomib (+bort) and CRISPR PSMG2 cells by western blots. **(I)** Measurement of the protein levels of various proteins degraded by the proteasome in RPMI-2650 and Detroit-562 control cells, control cells treated with bortezomib (+bort) and CRISPR PSMG2 cells by western blots. **(J)** Kaplan‒Meier analysis of overall survival in patients treated with bortezomib according to high or low levels of PSMG2 in the GSE9782 study. Statistical analysis was performed with Student's t test, *p < 0.05; **p < 0.01; ***p < 0.001.

The TCGA database was employed to examine the differential expression of PSMG2 in normal adjacent tissues and tumor tissues across multiple cancer types. Our findings showed a significant increase in PSMG2 expression within tumor tissues compared to normal tissues in most cancers examined (Figure 1A; acronyms defined in File S1). Notably, head and neck cancer exhibited a particularly marked difference in PSMG2 expression (p<0.001) (Figure 1A-B). Moreover, PSMG2 expression exhibited a progressive increase with advancing tumor stage, demonstrating significant differences between stage I and the later stages (II, III, and IV) (Figure 1C). Kaplan-Meier survival analysis was performed on the HNSCC TCGA dataset based on PSMG2 expression. Overall survival appeared to be worse in tumors with high PSMG2 expression. This trend was observed not only in the entire patient cohort (Figure 1D) but also when focusing on the subgroups of patients with the top 15% highest and bottom 15% lowest PSMG2 expression levels, whose survival outcomes were specifically evaluated (Figure 1E). These data suggest that PSMG2 is frequently overexpressed in tumor tissues compared to normal tissues, especially in head and neck cancer, and may be linked to a poorer patient prognosis.

In consequence, we explored the potential effects of decreasing PSMG2 expression on the tumorigenic capabilities of the RPMI-2650 and Detroit-562 head and neck cancer cell lines.

### The downregulation of PSMG2 inhibits proteasome activity

To study the effect of reduced PSMG2 expression on the tumorigenic properties of our HNSCC cell lines. First, we generated PSMG2 knockdown models using the CRISPR/Cas9 system. The clones R14 and R18 in RPMI-2650 cells and D5 and D7 in Detroit-562 cells were validated at the protein level by western blot analysis (Figure 1F) and the mRNA level by RT‒qPCR (Supplementary Figure S1A) and sequencing (Supplementary Figure S1B).

As PSMG2 is involved in the assembly of the 20S proteasome, we evaluated the alterations in this mechanism when PSMG2 expression is reduced. First, we observed that the PSMG1 protein level was diminished in the CRISPR PSMG2 cells compared to the control cells and the control cells treated with bortezomib (+Bort, an inhibitor of the proteasome) for both cell lines (Figure 1G). Moreover, PSMA5 and PSMA7/8 protein levels were reduced upon the downregulation of PSMG2 compared with those of the control cells of both cell lines (Figure 1G), as well as the protein level of PSMB1 in the Detroit-562 cells compared to the control cells (Figure 1G). Consequently, the level of ubiquitinated proteins increased in the CRISPR PSMG2 cells, as also observed in the controls treated with bortezomib in both cell lines (Figure 1H).

Additionally, we measured some target proteins that are degraded by the proteasome, such as HIF1α, CYCD1 and phospho-β-catenin. The levels of these proteins increased in the PSMG2- downregulated cells, as also observed in the controls treated with bortezomib in both cell lines (Figure 1I). These results showed that the downregulation of PSMG2 affects the assembly of the 20S proteasome and its activity, which triggers the accumulation of ubiquitinated proteins.

According to the relationship between PSMG2 and the proteasome, we studied the possible correlation between the response to proteasome inhibitor drugs and the expression level of PSMG2. The cytotoxic effects of several proteasome inhibitor drugs (bortezomib, ixazomib, and MG132), all of which target the 20S proteasome subunit, were evaluated in PSMG2 knockdown cells using IC50 cytotoxic assays. The Detroit-562 cell line, with low PSMG2 (CRISPR) cells were more sensitive to the drug effects than control cells (Supplementary Figure S2), however we did not observe variations in the RPMI-2650 cell line. Then, we analyzed the GSE9782 study which included patients with multiple myeloma in phases 2 and 3 treated with bortezomib. The survival of patients with high expression levels of PSMG2 was lower than that of patients with low expression levels of this gene (Figure 1J). These results suggest that the downregulation of PSMG2 could be a good prognostic and predictive biomarker for the response to treatment with proteasome inhibitor drugs.

However, due to the heterogeneity of HNSCC tumors, PSMG2 might be a good marker for the activity of proteasome inhibitors only in a subset of tumors, since only one cell line is more sensitive to proteasome inhibitors. Additionally, it can be a good marker in other tumors, such as multiple myeloma.

### ER stress and apoptosis are activated when PSMG2 is downregulated

**Figure 2:**
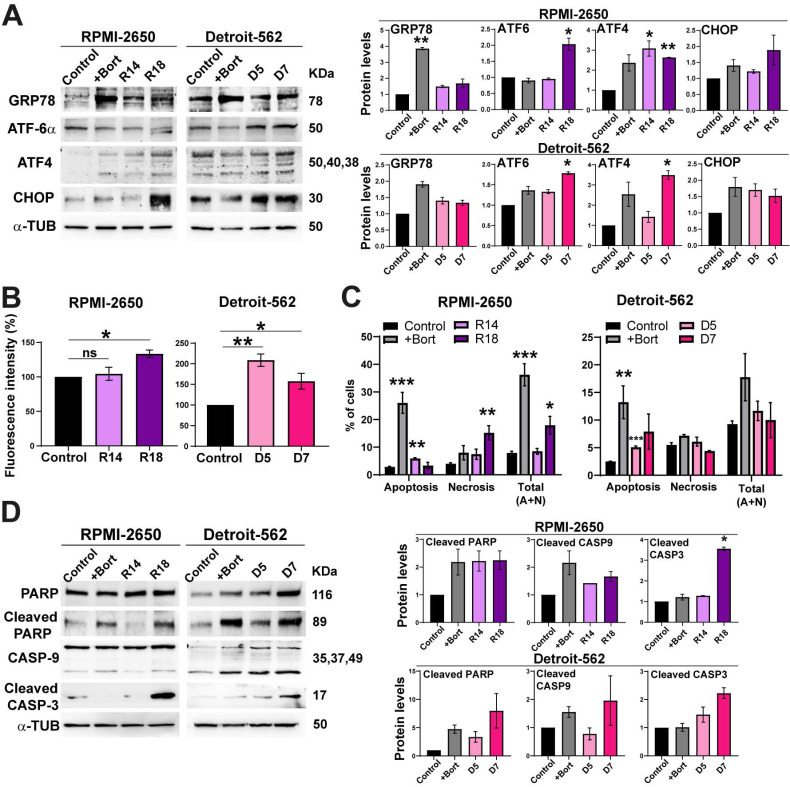
** Inhibition of the proteasome caused by the downregulation of PSMG2 induces ER stress and apoptosis. (A)** Measurement of the levels of proteins activated under ER stress in RPMI-2650 and Detroit-562 control cells, control cells treated with bortezomib (+bort) and CRISPR PSMG2 cell lines by western blots. **(B)** Percentage of fluorescence intensity of molecule H2DCFA to analyze the generation of ROS. **(C)** Percentage of cells in apoptosis, necrosis, and the combined total of apoptosis and necrosis in RPMI-2650 and Detroit-562 control cells, bortezomib-treated cells (+bort) and CRISPR PSMG2 cell lines by FACS. **(D)** Measurement of the levels of proteins implicated in the process of apoptosis in RPMI-2650 and Detroit-562 control cells, control cells treated with bortezomib (+bort) and CRISPR PSMG2 cell lines by western blots. *Left*, representative images of western blot analysis are shown; *right*, protein levels were quantified and normalized according to α-tubulin levels and to the control. The mean of a minimum of 3 independent experiments performed in triplicate ± standard error is represented in all experiments. Statistical analysis was performed with Student's t test, ns (or not labelled): non-significant, * p < 0.05, ** p < 0.01, *** p < 0.001. Abbreviations (applies to all figures): C/Control (parental cell line), +Bort (parental treated with bortezomib), R14 (RPMI CRISPR's PSMG2 14), R18 (RPMI CRISPR's PSMG2 18), D5 (Detroit CRISPR's PSMG2 5), D7 (Detroit CRISPR's PSMG2 7).

Inhibition of the proteasome causes an accumulation of proteins that triggers endoplasmic reticulum stress. The endoplasmic reticulum (ER) is responsible for making and modifying approximately one-third of cellular proteins 38. Stress in cells that affects this cell organelle disrupts protein processing and induces the unfolded protein response (UPR) 39. Glucose-regulated protein 78 (GRP78) binds to the hydrophobic domain of some proteins, located in the ER lumen, and protects against protein misfolding. In response to ER stress, GRP78 dissociates from these proteins, and different mechanisms are activated. ATF-6α is one of these proteins that is cleaved in the Golgi apparatus, translocates into the nucleus and activates gene transcription 40. Another of these proteins is PERK, which phosphorylates and activates the eI2F factor that activates ATF4 and the homologous protein C/EBP- (CHOP) 40. These proteins were analyzed to determine the existence of ER stress with PSMG2 downregulation. In both cell lines, we observed in general an increase in the protein levels of GRP78, ATF4, ATF-6α and CHOP in CRISPR clones and in the control cells treated with bortezomib compared to the control cells untreated (Figure 2A).

Moreover, ER stress can induce an increase in reactive oxygen species (ROS) 41. For this reason, we measured the fluorescence intensity of the carboxy-H2DCFDA molecule to detect alterations in the generation of ROS. The RPMI-2650 cell line showed a significant increase in fluorescence in clone R18 compared to the control (Figure 2B). In the Detroit-562 cell line, we observed a significant increase in the fluorescence intensity in both CRISPR clones (Figure 2B). These results indicate that the downregulation of PSMG2 causes ER stress and consequently increases the generation of reactive oxygen species (ROS).

When the cell cannot restore ER functionality, pathways such as apoptosis are triggered to eliminate damaged or dysfunctional cells 41. Cell death was analyzed using annexin V and propidium iodide markers by flow cytometry, allowing us to distinguish between apoptosis and necrosis. In both cell lines, we observed a significant increase in the percentage of apoptotic cells in the CRISPR clones (R14 and D5) and in the bortezomib-treated control cell line compared to the control cells. Regarding necrosis, the CRISPR clone R18 showed a significant increase in the percentage of necrotic cells (Figure 2C). Notably, the combined analysis of apoptosis and necrosis revealed an overall increase in cell death in the CRISPR clones compared to the control cells. In both cell lines, we observed an increase in cleaved PARP, CASP-9 and CASP-3 in CRISPR clones and in the bortezomib-treated controls (Figure 2D). These data suggest that the reduction in PSMG2 levels triggers an increase in apoptosis by increasing ER stress.

### Low levels of PSMG2 increase cytoprotective autophagy

**Figure 3:**
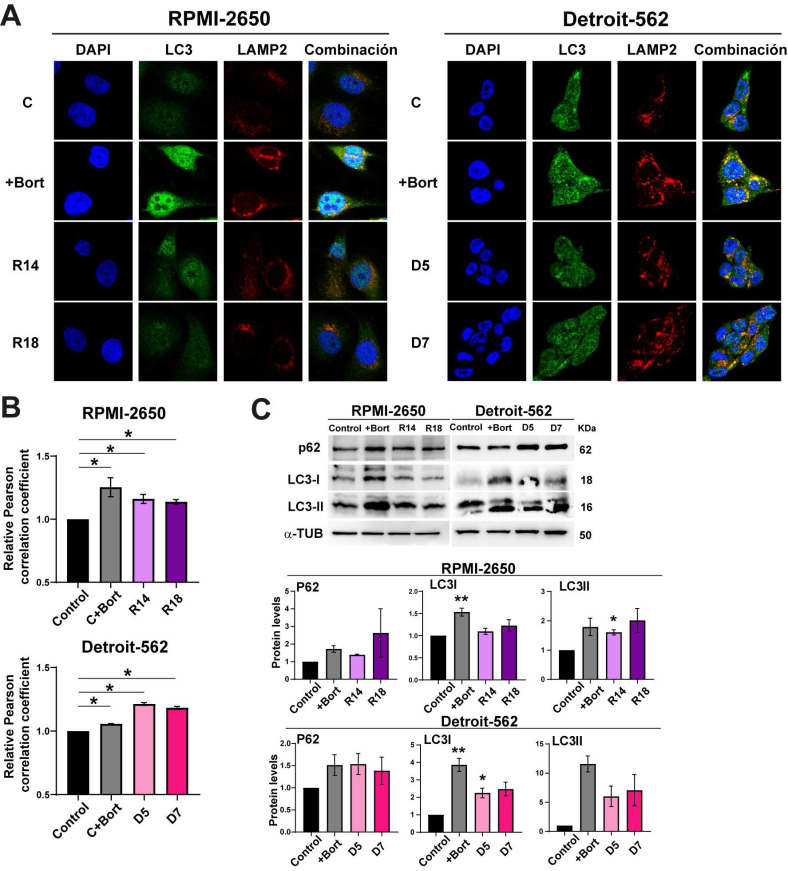
** The downregulation of PSMG2 increases autophagy as a compensatory mechanism. (A)** Colocalization of the autophagosome marker LC3 (green) and the lysosomal marker LAMP2 (red) was observed by immunofluorescence in control cells, control cells treated with bortezomib (+Bort) and CRISPR PSMG2 cells in RPMI-2650 and Detroit-562 cells. DAPI nuclear staining is shown in blue. **(B)** Quantification of colocalization between LC3 and LAMP2 by the Pearson correlation coefficient. The average and standard error of at least 200 cells in each condition are shown. **(C)** Measurement of the levels of proteins implicated in autophagy in RPMI-2650 and Detroit-562 control cells, control cells treated with bortezomib (+bort) and CRISPR PSMG2 cell lines by western blots. *Left*, representative images of western blot analysis are shown; *right*, protein levels were quantified and normalized according to α-tubulin levels and to the control. The mean of a minimum of 3 independent experiments performed in triplicate ± standard error is represented in all experiments. Statistical analysis was performed with Student's t test, * p < 0.05, ** p < 0.01, *** p < 0.001.

Inhibition of the proteasome causes apoptosis increasing ER stress, but also the activation of compensatory mechanisms to maintain cellular homeostasis. One of these processes is the lysosome-autophagy (ALP) degradation pathway, which is responsible for approximately 10-20% of protein degradation 42, 43. Effective autophagy requires fusion of the autophagosome with lysosomes; therefore, we analyzed autophagy using co-immunofluorescence staining of the autophagosome marker LC3 II and the lysosomal marker LAMP-2 (Figure 3A). We found that bortezomib treatment as a control induced autophagy in both cell lines (Figure 3A). As well, the downregulation of PSMG2 increased autophagy (Figure 3A). We measured the Pearson correlation coefficient of the colocalization of LC3 and LAMP2 using ImageJ JacoP software. We observed an increase in this coefficient in the CRISPR PSMG2 cells and bortezomib-treated controls in both cell lines compared to the controls (Figure 3B).

One of the proteins that connects the proteasome and autophagy is P62, which is a substrate for autophagy 42, 43. P62 (SQSTM1) can bind to ubiquitinated proteins to facilitate their degradation in the autophagosome. This process could limit the toxicity of undegraded proteins due to proteasome inhibition. Higher amounts of LC3-II correlate with an increase in the autophagy process. LC3-I is a substrate of the proteasome; therefore, the inhibition of the proteasome could cause the accumulation of this protein. Inhibition of the proteasome causes an increase in the phosphorylation of P62 at Ser405 and Ser409 and its transcription 44, 45. Therefore, we analyzed LC3 and P62 by Western blotting (Figure 3C). We observed that the protein levels of P62, LC3-I and LC3-II increased in the controls treated with bortezomib and in the CRISPR PSMG2 cells in both cell lines (Figure 3C). These results confirm the activation of autophagy in response to the downregulation of PSMG2.

### Inducing ER stress in HNSCC cell lines caused the activation of autophagy and apoptosis

According to previous results, the reduction of PSMG2 caused an increase of apoptosis and autophagy process. To study whether these effects are caused by increases in ER stress, we treated cells with Brefeldin-A (BFA), an inductor of ER stress in parental cell lines, and TUDCA, an inhibitor of ER stress in control and CRISPRs of PSMG2.

**Figure 4:**
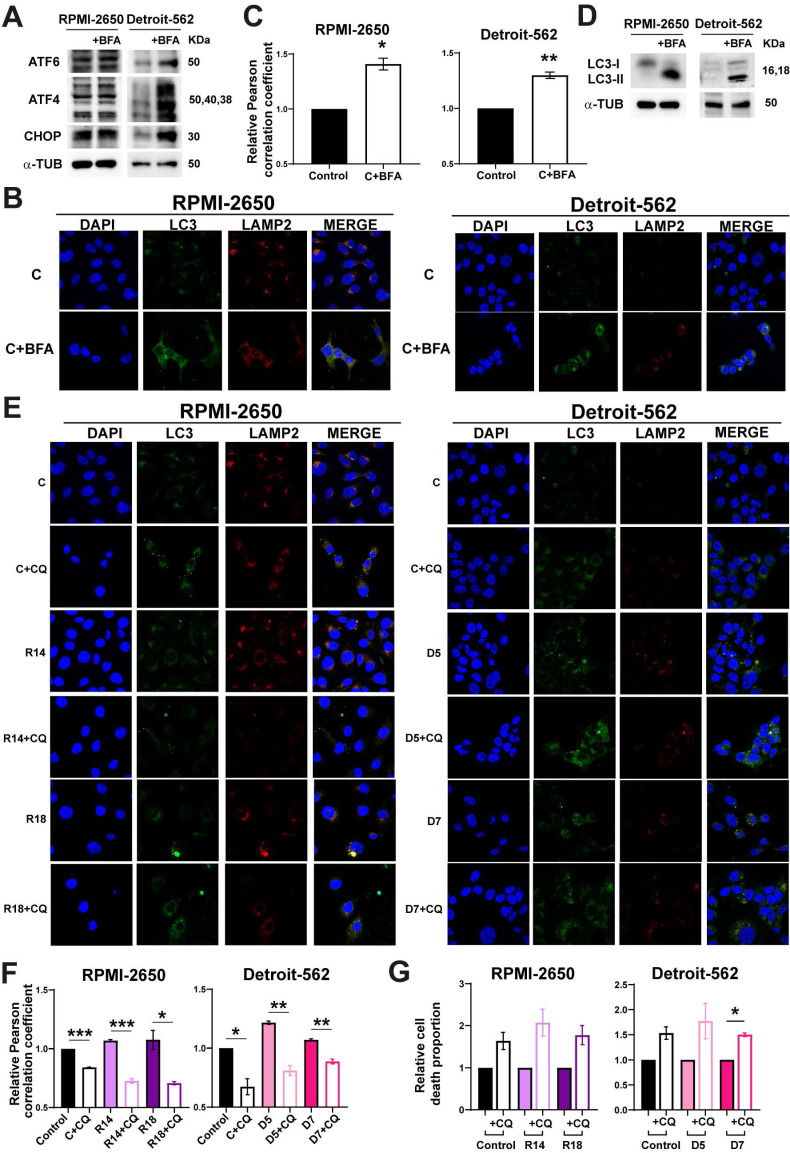
** (A-D) Brefeldin-A increased the autophagy process in RPMI-2650 and Detroit-562 cell lines. (A)** Measurement of the levels of proteins implicated in ER stress in RPMI-2650 and Detroit-562 control cells and control cells treated with Brefeldin-A (+BFA) cell lines by western blots. **(B)** Colocalization of the autophagosome marker LC3 (green) and the lysosomal marker LAMP2 (red) was observed by immunofluorescence in control cells and control cells treated with Brefeldin-A (+BFA) cell lines. DAPI nuclear staining is shown in blue. **(C)** Quantification of colocalization between LC3 and LAMP2 by the Pearson correlation coefficient. The average and standard error of at least 200 cells in each condition are shown. **(D)** Measurement of the levels of LC3 in RPMI-2650 and Detroit-562 control cells and control cells treated with Brefeldin-A (+BFA) cell lines. **(E-G) Chloroquine effect in CRISPRs of PSMG2 in RPMI-2650 and Detroit-562 cell lines. (E)** Colocalization of the autophagosome marker LC3 (green) and the lysosomal marker LAMP2 (red) was observed by immunofluorescence in control cells, CRISPRs PSMG2 and control and CRISPRs cells treated with chloroquine (+CQ) cell lines. DAPI nuclear staining is shown in blue. **(F)** Quantification of colocalization between LC3 and LAMP2 by the Pearson correlation coefficient. The average and standard error of at least 200 cells in each condition are shown. **(G)** Percentage of death cells in RPMI-2650 and Detroit-562 control cells, CRISPRs PSMG2 and control and CRISPRs cells treated with chloroquine (+CQ) cell lines by FACS. The mean of a minimum of 3 independent experiments performed in triplicate ± standard error is represented in all experiments. Statistical analysis was performed with Student's t test, * p < 0.05, ** p < 0.01, *** p < 0.001.

First, RPMI-2650 and Detroit-562 cells were treated with Brefeldin-A to induce ER stress. We observed the treatment with Brefeldin-A (BFA) caused an increase of the proteins involved in ER stress (Figure 4A). Autophagy was analyzed by co-immunofluorescence and Western-blot. The co-localization of LC3 and LAMP2 proteins had a significative increase when cells were treated with BFA in both cell lines (Figure 4B-C). Furthermore, we observed an increase in the protein level of LC3 in cells treated with BFA compared to the controls in both cell lines (Figure 4D).

To study whether ER stress is the cause of cellular death observed when PSMG2 levels are reduced, we treated cells with TUDCA. We observed that control and CRISPRs of PSMG2 had a reduction in the percentage of apoptotic, necrotic and total cell death in RPMI-2650 and in apoptotic cells in Detroit-562 (Figure S3A). Total cell death refers to the combined percentages of both apoptosis and necrosis, highlighting the overall decrease in cell death when treated with TUDCA. On the other hand, we analyzed cell death in BFA-treated cells and observed that the induction of ER stress led to a higher proportion of apoptotic and necrotic cells (Fig. S3B). Additionally, cleaved PARP levels increased under these conditions in both cell lines (Fig. S3C). In turn, these results suggest that ER stress could be one of the causes of the increased apoptosis observed upon PSMG2 reduction. To test the relationship between autophagy and apoptosis process when proteasome was inhibited, we employed chloroquine (CQ), an inhibitor of autophagy process. The treatment with CQ caused the reduction of the co-localization of LC3 an LAMP2 in controls and CRISPRs of PSMG2 treated with CQ (Figure 4E-F). Cell death was studied by flow cytometry analyzing Annexin-V and propidium iodide markers to distinguish between apoptotic, necrotic and total cell death. We observed that both controls and PSMG2 CRISPRs treated with CQ increased the cell death (Figure 4G). These results suggest that autophagy in our model acts as a protective mechanism, mitigating cellular stress and enhancing cell survival.

### Effect of PSMG2 downregulation on the tumorigenic properties of HNSCC cell lines

**Figure 5:**
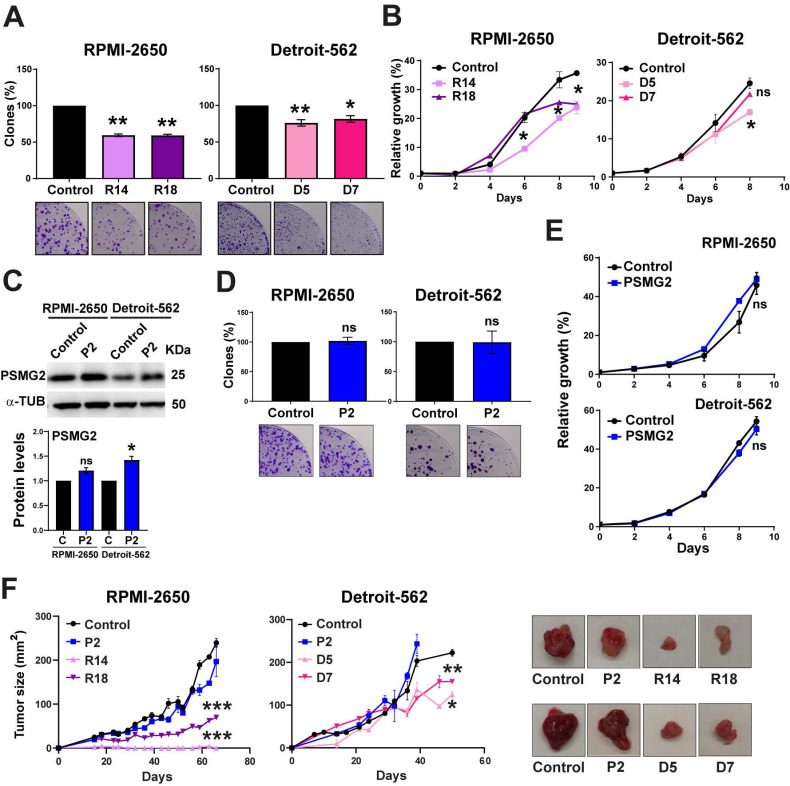
** Downregulated PSMG2 decreases tumorigenesis in HNSCC cell lines *in vitro* and* in vivo*. (A)** Clonogenic assay of RPMI-2650 and Detroit-562 control and PSMG2 CRISPR cell lines. Cells were seeded at low density, and after 15 days, colonies were counted. Representative images are shown. **(B)** Growth curves of RPMI-2650 and Detroit-562 control and PSMG2 CRISPR cell lines. **(C)** Validation of PSMG2 overexpressed in RPMI-2650 and Detroit-562 cell lines by Western-blot. **(D)** Clonogenic assay of RPMI-2650 and Detroit-562 control and PSMG2 overexpressed cell lines. Cells were seeded at low density, and after 15 days, colonies were counted. Representative images are shown. **(E)** Growth curves of RPMI-2650 and Detroit-562 control and PSMG2 overexpressed cell lines. **(F)** Tumor growth in xenografts from RPMI-2650 and Detroit-562 control, PSMG2 overexpressed and CRISPR PSMG2 cell lines. Cells were injected into nude mice (N=6), and tumor size was measured weekly. Graphs represent the tumor size (mean ± SEM). Representative images of tumor size are shown. The mean of a minimum of 3 independent experiments performed in triplicate ± standard error is shown in all experiments. Statistical analysis was performed with Student's t test, ns: non-significant, * p < 0.05, ** p < 0.01.

To study the effect of reduced PSMG2 expression on the tumorigenic properties of our HNSCC cell lines, we performed some functional assays, growth curve and clonability assay. We observed that the PSMG2 knockdown cells formed fewer colonies (Figure 5A) and grew slower than the control cells of both HNSCC cell lines (Figure 5B). On the other hand, we overexpressed the level of PSMG2. The overexpression PSMG2 was validated by Western-blot analysis (Figure 5C) and RT-q-PCR (Figure S4A). The overexpression of PSMG2 did not cause any alterations in the proliferation properties of RPMI-2650 and Detroit-562 cell lines (Figure 5D-E). The absence of effects on tumor properties upon overexpression of PSMG2 may be due to the saturated expression levels of PSMG2 in our cell lines. As shown in the bioinformatic analysis, tumors from patient samples exhibit elevated levels of this gene, suggesting that most head and neck cancer cell lines may already have saturated levels of PSMG2. Regarding the *in vivo* assays, it was shown that PSMG2 CRISPRs formed smaller tumors and grew more slowly than the control cells (Figure 5F). Overexpression of PSMG2, however, resulted in a similar behavior to the control cells (Figure 5F). These findings further support that reducing PSMG2 levels decreases the tumorigenic properties of HNSCC cell lines both *in vitro* and *in vivo*.

### Effect of the reduction in PSMG2 levels on the stemness properties of HNSCC cell lines

**Figure 6:**
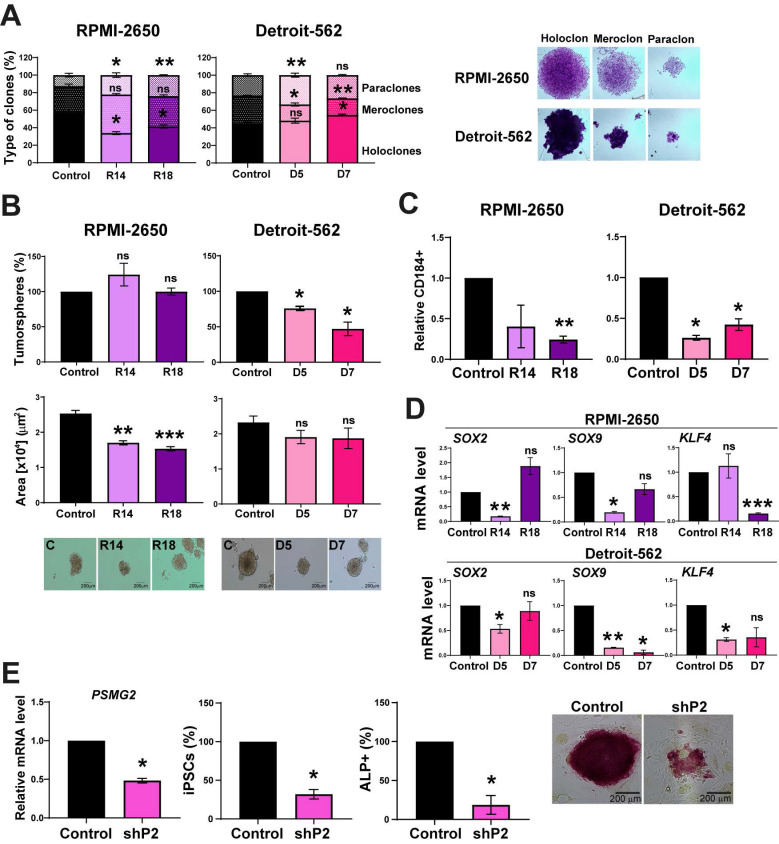
** The reduction in PSMG2 diminishes stemness and affects the cellular reprogramming of MEFs. (A)** Percentages of holoclones, meroclones and paraclones generated by RPMI-2650 and Detroit-562 control and CRISPR PSMG2 cell lines seeded at low density over 15 days. **(B)** Percentages of tumorspheres formed from the whole population of RPMI-2650 and Detroit-562 control and CRISPRs PSMG2 cell lines. Quantification of the tumorsphere area. Representative images of the tumorspheres are shown (scale bars: 200 µm). **(C)** Quantification of the number of CD184 positive cells in RPMI-2650 and Detroit-562 control and CRISPR PSMG2 cell lines by FACS. **(D)** Measurement of *SOX2, SOX9* and* KLF4* expression levels by RT‒qPCR in RPMI-2650 and Detroit-562 control and CRIPSRs of PSMG2 cell lines. Graphs represent mRNA levels in the CRISPR PSMG2 cells normalized to the mRNA levels of the control cells. **(E)** Generation of iPS cells from MEFs comparing the control and shRNA of PSMG2 (shPSMG2). Validation of the reduction in PSMG2 in the shPSMG2 cells with respect to the controls. Percentage of iPS cells formed in the control and shPSMG2 cells. Percentage of iPS cells positive for alkaline phosphatase assay and representative images of this assay. The mean of a minimum of 3 independent experiments performed in triplicate ± standard error is shown in all experiments. Statistical analysis was performed with Student's t test, ns: non-significant, * p < 0.05, ** p < 0.01.

PSMG2 forms part of the proteasome, which has been previously related to stemness 46-50. To explore the effect of the downregulation of PSMG2 on the stemness capability of the cells, we performed a clonability assay to measure the phenotypes of the clones as described in 51-53. We found that the PSMG2 CRISPR cells formed a lower percentage of holoclones (clones enriched in CSCs and able to regenerate the culture) and a higher percentage of paraclones (clones enriched in differentiated tumor cells and unable to regenerate the culture) compared to the control cells of both cell lines (Figure 6A). We also observed that the PSMG2-reduced cells formed smaller tumorspheres in the RPMI-2650 group and a lower number of tumorspheres in the Detroit-562 group (Figure 6B). In addition, we analyzed the positive cells for CD184, a CSCs surface marker. In previous results of the group, we validated the percentage of cells positive for some markers as CD44, CD166 in RPMI-2650 and Detroit-562 54. All the populations were positive to CD44 and CD166, so they could not be good markers for these cell lines 54. In addition, it was validated the effect of CD184 by in vivo assay and we observed that the tumors formed from CD10 +, CD184 + or CD166 + cells grew faster and more aggressively than the tumors formed from negative cells 54. Therefore, CD184 is a good marker related to cancer stem-like cells in our cell lines. Downregulated PSMG2 cells showed a lower percentage of CD184 positive cells in both cell lines (Figure 6C). These data confirm the functional role of PSMG2 and CSCs in HNSCC.

In cells with lower PSMG2 levels, the mRNA expression of some CSC markers, such as *SOX2, SOX9* and* KLF4,* was downregulated (Figure 6D). These data indicate that the downregulation of PSMG2 reduces stemness in HNSCC cell lines, probably by reducing pluripotency transcription factors.

To study the role of PSMG2 in the dedifferentiation process, we transfected MEFs with 4 Yamanaka factors (*OCT4, KLF4, SOX2* and* MYC*) and the shRNA scramble (control) or shRNA PSMG2 (shPSMG2) to analyze the possible alterations in reprogramming. We observed that the downregulation of PSMG2 caused a reduction in the number of iPS cells formed with shPSMG2 compared with the control (Figure 6E). Moreover, the number of alkaline phosphatase (ALP)-positive cells, a marker commonly used to identify induced pluripotent stem cells (iPSCs) as part of the reprogramming process from MEFs, was reduced in the shPSMG2 cells compared to the control cells (Figure 6E). This result suggests that PSMG2 is involved in the dedifferentiation process and that the reduction in the level of PSMG2 affects reprogramming MEFs.

On the other hand, the overexpression of PSMG2 did not cause alterations in the stemness properties of the RPMI-2650 and Detroit-562 cell lines (Figure S4), probably because the cells could have saturated expression of PSMG2 and proteasomal activity and, consequently, do not respond to further increases in PSMG2 levels.

## Discussion

PSMG2 is involved in the assembly of the 20S proteasome, forming a heterodimer with PSMG1. The assembly process begins with the formation of the α ring supported by the complex of the heterodimeric chaperones PSMG1-PSMG2 and PSMG3-PSMG4 28-32. The exact mechanism of α ring assembly is unknown. The downregulation of PSMG3 and PSMG4 caused a reduction in α ring formation 29, 31. Moreover, the PSMG1-PSMG2 heterodimer acts only on the α ring and is responsible for preventing its dimerization, which could cause arrest in the biogenesis of the 20S proteasome subunit 27. This heterodimer interacts directly with the α5 and α7 subunits 28. The reduction in PSMG1 or PSMG2 caused the accumulation of α ring dimers 28. The proteasome is a cellular multienzymatic machinery responsible for the degradation of nonfunctional, misfolded, damaged and/or foreign proteins 27. The normal activity of the proteasome is very important in cells to maintain cellular homeostasis 27. It plays an essential role in numerous cellular processes, such as signal transduction, cell death, and protein quality control 27.

The differential expression of PSMG2 in various databases indicates that this gene is upregulated in tumors compared to normal tissue. Specifically, in HNSCC, PSMG2 is significantly overexpressed in tumor tissue, with its expression increasing as the tumor stage progresses and correlating with poorer overall survival, suggesting its potential oncogenic role. Consistent with this, downregulation of PSMG2 in the HNSCC cell lines RPMI-2650 and Detroit-562 led to a significant reduction in proliferation in vitro and tumor growth in vivo, further supporting its potential as a prognostic biomarker and therapeutic target for these tumors.

An extensive analysis of PSMG2 interactions was performed using the BioGRID and IntAct databases. Genes that were common to both databases were selected for subsequent functional analysis: Gene Ontology (GO) enrichment analysis (Figure S4C) and Reactome pathway analysis (Figure S4D). The results revealed that, as expected, the ubiquitin-proteasome system (UPS) was strongly associated, along with other processes such as the cell cycle, apoptosis, and NF-κB signaling.

To elucidate the potential mechanisms underlying PSMG2's oncogenic function, we studied its role in proteasome-mediated protein degradation. We observed a reduction in PSMG1 levels when PSMG2 was downregulated. The lack of one of these two proteins causes a reduction in the other, as they are not stable without forming a heterodimer. These proteins have a very short half-life, approximately 40 minutes 28. We also observed a reduction in the protein levels of α5 and α7 subunits (PSMA5 and PSMA7/8) in the downregulation of PSMG2. Most likely, the α ring cannot be formed correctly due to the lack of the PSMG1-PSMG2 heterodimer, and α ring dimers are generated without the ability to follow the assembly process 28. When the levels of PSMG2 were reduced, we observed an accumulation of ubiquitinated proteins, indicating that the proteasome was deficient and not fully functional and that the proteins could not be degraded when PSMG2 was downregulated. Previously, it had been reported that reduced PSMG1 levels also caused an increase in short-lived proteins such as p35 and HIF1α, accumulating mainly in their ubiquitinated form 55. Due to the spread spectrum of proteins that are degraded by the proteasome, a large number of cellular responses and signaling pathways are expected to be affected by its inhibition.

Inhibition of the proteasome has emerged as a potential cancer therapy by disrupting protein degradation, leading to cellular stress and apoptosis. While proteasome inhibitors have shown success in certain type of cancers, their effectiveness can be influenced by compensatory mechanisms such as autophagy and ER stress 56. Bortezomib was approved for the treatment of multiple myeloma. The GSE9782 study, which included multiple myeloma patients in phases 2 and 3 treated with bortezomib, allowed us to obtain a gene expression profile. The analysis of PSMG2 expression and response to treatment showed that patients with lower levels of PSMG2 treated with bortezomib had better survival than patients with high levels. Furthermore, the downregulation of PSMG2 caused an increase in chemosensitivity to treatment with bortezomib, ixazomib and MG-132 in at least one HNSCC cell line. These responses may be linked to the role of PSMG2 in the regulation of proteasome activity and cellular stress responses. The reduction of PSMG2 could disrupt these protective mechanisms, making cancer cells more vulnerable to therapeutic agents. Therefore, PSMG2 could be used as a prognostic and predictive marker of response to treatment with proteasome inhibitor drugs.

Inhibition of the proteasome exposes cells to endoplasmic reticulum (ER) stress by preventing the removal of misfolded proteins 56. Approximately one-third of all cellular proteins are synthesized and modified in the ER 38. Peptides are folded and acquire their tertiary conformation within the lumen of this organelle 38. Therefore, stimuli that disrupt the processing of these peptides can lead to an accumulation of misfolded or unfolded proteins, causing ER stress and inducing the unfolded protein response (UPR) 39. This mechanism is activated to restore ER activity and is characterized by increased degradation of misfolded proteins by the proteasome, decreased global protein synthesis, and transcriptional and protein upregulation of chaperones and foldases 57. In our results, we observed that proteins activated under ER stress, such as GRP78 and ATF-6, were increased in PSMG2-downregulated cells, confirming that ER stress was occurring. Moreover, the generation of reactive oxygen species (ROS) was increased when PSMG2 was reduced, confirming the increase in cellular stress. If this stress cannot be restored, escape pathways such as apoptosis are activated to re-establish cellular homeostasis 56. In our case, apoptosis was analyzed using both flow cytometry and Western blot showing that the reduction in PSMG2 increased apoptosis. Moreover, the employ of TUDCA, an inhibitor of ER stress, in our conditions reduced the death cells in RPMI-2650 suggesting that the activation of apoptosis is produced by the ER stress.

Proteasome inhibition also triggers activation of the lysosome-autophagy degradation pathway as a compensatory mechanism to diminish proteotoxic stress and avoid cell death 42. This activation could be produced by the induction of ER stress because of the treatment with BFA, an inductor of ER stress, in our HNSCC cell lines caused the activation of autophagy process. The p62 protein can act as a mediator signal between the UPS and autophagy. This molecule is responsible for transporting them to their corresponding degradation destination, the proteasome or autophagy 58. In our results, we observed an increase in P62, LC3-I and LC3-II protein levels when PSMG2 was reduced. Given that p62 is also degraded via autophagy, its accumulation suggests that while autophagy is active, the lysosomal degradation step may be compromised or overwhelmed due to the high protein load. This aligns with previous studies demonstrating that proteasomal inhibition triggers compensatory autophagy, which may not be sufficient to handle excessive protein accumulation. LC3-I is a substrate of the proteasome, which explains its accumulation. The colocalization of LC3 and LAMP2, which confirms the fusion of autophagosomes with lysosomes, was higher in the downregulation of PSMG2. However, despite this increased co-localization, the accumulation of LC3-II and p62 suggests that the final degradation step may be impaired or that the autophagic system is overloaded and unable to clear all accumulated substrates efficiently. This behavior is also observed with bortezomib treatment, reinforcing the idea that proteasomal inhibition by downregulation PSMG2 triggers a compensatory but possibly insufficient autophagic response. The relationship between autophagy and cell death was examined through chloroquine treatment to determine whether these mechanisms are interconnected or occur independently. In our model, inhibition of autophagy resulted in a significant increase in cell death, indicating that autophagy acts as a protective mechanism under these conditions.

In terms of stem cell properties, we observed that the downregulation of PSMG2 decreases stemness. In addition, we observed a lower number of positive cells for the markers CD184. CD184 marker has previously been proposed as markers of CSCs in HNSCC 54, 59-61. The reduction in PSMG2 levels in MEFs caused a reduction in the efficiency of iPS formation, which could mean a partial inhibition of cell reprogramming. This result could be because the reduction in PSMG2 alters the activity of the proteasome, an essential mechanism that acts as a regulator of pluripotency 46-50, 62-66. In mESCs, the activity of the proteasome was produced via tissue-specific transcription factors and/or RNA polymerase II to inhibit the transcription of these targets. The inhibition of the proteasome or the silencing of proteasome subunits improved the binding of specific transcription factors and activated RNA polymerase II to start the differentiation process 49. Furthermore, the 20S proteasome plays a crucial role in degrading proteins and regulatory complexes involved in the maintenance of transcriptional elongation. This finding suggests that the proteasome acts as a transcriptional silencer in mESCs to preserve pluripotency 49. Silencing Psmd14/Rpn11, a deubiquitinating enzyme expressed in mESCs, decreases reprogramming efficiency and pluripotency, while its overexpression enhances the pluripotent state and inhibits differentiation 50. In hESCs, the proteasome could also play an essential role in regulating the dedifferentiation process. The expression of one crucial subunit, PSMD11/RPN6, is high in hESCs and iPSCs but decreases during cellular differentiation, resulting in a reduction in proteasome assembly and activity 67. Inhibition of the proteasome in pluripotent cells downregulates stemness-related genes (*OCT4, SOX2, NANOG* and *c-MYC*) and upregulates differentiation markers (*FGF5* and *GATA4*) 67. Moreover, MG-132 treatment at low concentrations over a long period of time (3 and 14 days) 50 and specific proteasome inhibitors, such as UK101 and PK957, caused the inhibition of cellular reprogramming and induced the loss of self-renewal 68, which is consistent with our results.

## Conclusions

The reduction in PSMG2 altered the activity of the proteasome, which was decreased. In the process of tumorigenesis, the inactivity of the proteasome induces ER stress due to the accumulation of misfolded and ubiquitinated proteins. ER stress activated ROS production and apoptosis. In addition, the inhibition of the proteasome generated an increase in autophagy as a compensatory mechanism to maintain cell homeostasis. Regarding stem cell properties, inhibition of the proteasome reduced the number of tumorspheres and the transcriptional expression of genes related to the stem cell phenotype, such as *SOX2* and *SOX9*. This observed phenotype could be due to the involvement of the proteasome in the pluripotency and differentiation process. In addition, the reduction in PSMG2 caused low efficiency of the cellular reprogramming of MEFs. Therefore, the decrease in PSMG2 levels could inhibit the dedifferentiation process.

## Materials and methods

### Cell culture

RPMI-2650 and Detroit-562 cell lines were obtained from the ECACC commercial repository. No further authentication was conducted by the authors. Cells were negative for mycoplasma. RPMI-2650 and Detroit-562 cell lines were maintained in DMEM (Gibco) with 10% fetal bovine serum (FBS) (Gibco), penicillin, streptomycin and fungizone (Sigma). MEFs (mouse embryonic fibroblasts) were maintained in DMEM (Gibco) with 10% fetal bovine serum (FBS) (Gibco), penicillin and streptomycin (Sigma). Feeder layer (SNL) was maintained in DMEM (Gibco) with 15% fetal bovine serum (FBS) (Gibco), penicillin and streptomycin (Sigma).

### Retroviral infection

Retroviral vectors and gene transfer were performed as previously described in 69.

### CRISPR/Cas9 to generate PSMG2 knockout

An sgRNA targeting the PSMG2 sequence TTCCGGTACCTACTTACACC (exon 5) was used to generate knockout models from VectorBuilder (pLV[CRISPR]-hCas9:T2A:Puro-U6>hPSMG2[gRNA#6615]). First, we infected the cells with virus containing PSMG2-sgRNA and drug selection. Then, the cells were isolated by single-cell sorting by FACS Jazz (BD Biosciences) in 96-well plates. One month later, each well that grew was amplified and validated by western blot analysis. The selected CRISPRs were sequenced in the Genomics and Sequencing service at IBiS.

### RT‒qPCR

Total RNA from cell lines was extracted and purified using the ReliaPrepTM RNA Tissue Miniprep System (Promega), and reverse transcription was performed with 3 µg of mRNA using the High Capacity cDNA Reverse Transcription kit (Life Technologies) according to the manufacturer's instructions. The PCR mixture (10 µL) contained 2 µL of the reverse transcriptase reaction product diluted 1:10, 2.5 µL of water, 5 µL of GoTaqR Probe qPCR Master Mix (Promega) and 0.5 µL of the appropriate TaqMan Assay (20X) (Applied Biosystems). We used the following probes: GAPDH (Hs03929097_g1) as an endogenous control, PSMG2 (Hs.PT.58.27209203.g), SOX2 (Hs01053049_s1), SOX9 (Hs01001343_g1) and KLF4 (Hs00358836_m1).

### Protein isolation and Western blot analysis

Western blots were performed as previously described elsewhere. Membranes were incubated with the following primary antibodies: anti-PSMG2 (1:1000; Abcam, ab172909), anti-PSMG1 (1:2000; CSB-PA018921LA01HU), anti-PSMA7/8 (1:1000; Santa Cruz Biotechnologies, sc-166761), anti-PSMA5 (1:1000; Santa Cruz Biotechnologies, sc-137240), anti-PSMB1 (1:1000; Santa Cruz Biotechnologies, sc-374405), anti-ubiquitinated proteins (1:500; GeneTex, GTX128826), anti-HIF-1α (1:200; Cayman Chemical, 10006421), anti-CYCD1 (1:1000; Santa Cruz Biotechnologies, sc-8396), anti-phospho-β-catenin (1:1000; Cell signaling #9561), anti-ATF-6α (1:1000; Santa Cruz Biotechnologies, sc-166659), anti-ATF4 (1:1000; Santa Cruz Biotechnologies, sc-390063), anti-GRP78 (1:1000; Santa Cruz Biotechnologies, sc-13539), anti-CHOP (1:1000; Cell signaling #2895), anti-PARP (1:1000; Cell signaling #9532), anti-cleaved Caspase-3 (Asp175) (5A1E) (1:1000; Cell Signaling #9664), anti-Caspase-9 (1:1000; Cell Signaling #9502), anti-LC3B (1:1000; Abcam, ab48394), anti-SQSTM1/p62 (1:10000; Abcam, ab109012) and anti-α-tubulin (Sigma T9026) as a loading control. Horseradish peroxidase-labeled rabbit anti-mouse (Abcam ab97046) and goat anti-rabbit (Abcam ab97051) secondary antibodies were used. The proteins were detected using an ECL detection system (Amersham Biosciences) and a Bio-Rad ChemiDoc Touch.

### Autophagy analyses

Cells were seeded onto glass coverslips, fixed with 4% paraformaldehyde for 20 minutes, and permeabilized with 0.5% Triton X-100 for 5 minutes. The coverslips were incubated with anti-LC3B (1 μg/mL; Abcam #ab48394) and anti-LAMP2 (1 μg/mL; Abcam #ab25631). Anti-rabbit Alexa Fluor 488 and anti-mouse Alexa Fluor 546 were used as secondary antibodies. The nuclei were counterstained with DAPI, and the slides were mounted with Prolong Gold Antifade (Life Technologies). The samples were visualized with a confocal ultraspectral microscope (Leica Stellaris 8) by sequential scanning of the emission channels. Autophagy was defined as colocalization of LAMP2 and LC3B. The mean intensity of the fluorescence was measured for a minimum of 200 cells per condition using ImageJ JaCoP Software. Statistical significance was calculated using Student's t test.

### Measurement of ROS generation

To detect reactive oxygen species (ROS), we used the compound carboxy-H_2_DCFDA. This molecule is not fluorescent, but in the presence of ROS, it is oxidized by the peroxidase enzyme and becomes fluorescent green 70. First, cells were seeded in 96-well plates to 80% confluence the next day. At 24 hours, medium without phenol red with H_2_DCFDA was added to several wells, leaving control wells without this molecule. The samples were incubated for 15 minutes at 37 °C. Finally, fluorescence was measured at 10-minute intervals by a CLARIOstar microplate reader.

### Apoptosis assay

To analyze the number of apoptotic cells, we employed the *Annexin V FITC Apoptosis Detection Kit.* A total of 1x10^6^ cells were trypsinized and resuspended in 200 μl of a mixture containing binding buffer (195 μl) and Annexin V (5 μl) and incubated for 15 minutes. After washing the cells with binding buffer, we suspended the cells in 100 µL of binding buffer (97.5 μl) and propidium iodide (2.5 μl). The cells were incubated for 5 minutes, and binding buffer was added. Finally, we analyzed the cells by FACS with a FACSCanto II cytometer (BD Biosciences). Experiments were repeated a minimum of three times independently in triplicate samples.

### Growth curve

For measurement of the proliferative capacity, 2.5x10^3^ (RPMI-2650) or 8x10^3^ (Detroit-562) cells were seeded in 12-well plates in triplicate. At 24 h (Day 0), cells were fixed with 0.5% glutaraldehyde (Sigma), and every 48 h, a curve point was fixed up to 10 days. Once all the points were collected, plates were stained with 1% crystal violet (Sigma). Then, the violet crystal was dissolved in 20% acetic acid (AppliChem), and the relative number of cells was quantified by measuring the absorbance of the violet crystal at 595 nm by an absorbance reader (Bio-Rad). The values were represented referring to Day 0.

### Clonogenic assay

For measurement of the ability of cells to form individual clones, 500 (RPMI-2650) or 5x10^3^ (Detroit-562) cells were plated in 10 cm plates in triplicate. Cells were fixed with 0.5% glutaraldehyde and stained with 1% crystal violet after 15 days. The number of colonies was counted, and types of clones were classified.

### Tumorsphere assay

A total of 1×10^4^ (RPMI-2650) or 2×10^4^ (Detroit-562) cells were seeded in triplicate in 24-well ultralow attachment plates (Costar) containing 1 mL of MammoCult basal medium (Stem Cell Technologies) with 10% MammoCult proliferation supplement, 4 μg/mL heparin, 0.48 μg/mL hydrocortisone, penicillin and streptomycin. After 5-10 days, depending on the cell line, the number of primary tumorspheres formed was measured using an inverted microscope (Olympus IX-71).

### Cytotoxic assay

A total of 1.5×10^4^ RPMI-2650 cells or 3×10^4^ Detroit-562 cells were seeded in 96-well plates. The cells were treated 24h later with different concentrations of the drugs: 1-0 μM Bortezomib, 10-0 μM ixazomib,10-0 μM MG132 and 1-0 μM Paclitaxel. After 96 h, the cells were stained with 0.5% crystal violet. Then, the violet crystal was solubilized in 20% acetic acid (Sigma) and quantified at 595 nm absorbance to measure the cell viability.

### Fluorescence-activated cell sorting (FACS) analysis

For FACS analysis, 1x10^6^ cells were trypsinized and suspended in 100 µL of PBS containing 2% FBS and 5 mM EDTA. Cells were blocked with 10 µL of human blocking reagent (Miltenyi Biotec) for 10 min at 4 °C. Then, the cells were incubated with 2 µL of anti-CD184-PE (Miltenyi Biotec #130-117-690) for 30 min at 4 °C. After the cells were washed twice with PBS-FBS-EDTA, they were suspended in 500 µL of the same buffer and analyzed by FACS with the FACSCanto II cytometer (BD Biosciences). Experiments were repeated a minimum of three times independently in triplicate samples.

### iPS generation assay

SNL cells and MEFs were used to generate iPS cells. First, PhoenixA cells were seeded in 10 cm diameter dishes and cultured for 24 hours. A retroviral infection of PhoenixA was performed with 80 μl PEI and 10 μg of one of the plasmids pMXs (Oct3/4, Sox2, Klf4, c-Myc and DsRed, this last one as a transfection control), followed by incubation for 20 hours. Lentiviral infection of HEK293-T cells was performed with 2 μg of pMD2G, 8 μg of psPAX2 and 10 µg of one of the following plasmids: pGreenZeo mNanog, control shRNA Plasmid-A (Santa Cruz Biotechnologies; sc-108060) and shRNA-PSMG2 (Santa Cruz Biotechnologies; sc-76033-SH). The mixture was incubated for 30 minutes and added dropwise to the cells. The sample was incubated for 24 hours. Medium from retrovirus- and lentivirus-producing cells was collected and filtered using a 0.45 µm acetate-cellulose filter. Polybrene was added to a final concentration of 4 µg/ml. Mixtures were made with different plasmids and added to MEFs previously seeded in a 6-well plate at 10^5^ cells per well. The cells were incubated for 24 hours, and the medium was changed. At 72 hours, 1700 infected MEFs were plated in 6-well plates containing a feeder layer of mitomycin-C-inactivated SNL cells (350,000 cells per plate). The next day, embryonic stem cell culture medium (ES medium) composed of DMEM with 15% FBS, 2 mM L-glutamine, 10^-4^ M nonessential amino acids, 10^-4^ M 2-mercaptoethanol, and 50 mg/ml penicillin and streptomycin solution were added to the wells. Eight days after the first iPS cell appeared, we performed the alkaline phosphatase assay. On Day 21, we counted Nanog-GFP-positive colonies under a fluorescence microscope.

### Alkaline phosphatase assay (ALP)

We used the Alkaline phosphatase detection kit (Sigma), which contains two reagents: Fast Red Violet solution (FVR) (0.8 g/L stock) and naphthol AS-BI phosphate solution (4 mg/mL) in AMPD buffer (2 mol/L), pH 9.5. First, cells were fixed with paraformaldehyde in 4% PBS for 1 minute and rinsed with PBS-0.1% Tween. The mixture of reagents in the ratio 2 (FVR):1 (naphthol):1 (water) was added to the wells and incubated for 15 minutes in the dark. The cells were washed with PBS-0.1% Tween, and the pink colonies were observed under an inverted microscope.

### Xenograft in nude mice

Tumorigenicity was assayed by the subcutaneous injection of 3×10^6^ RPMI-2650 cells or 2×10^6^ Detroit-562 cells into the right flanks of 4-week-old female athymic nude mice. Cells were suspended in 50 µL of Matrigel (Corning) prior to the injection. Animals were examined weekly until the tumor size was approximately 300 mm^3^, and mice were sacrificed. Tumors were extracted and stored at -80 °C. Tumor volume (mm^3^) was measured using calipers. All animal experiments were performed according to the experimental protocol approved by the IBIS and HUVR Institutional Animal Care and Use Committee (0309-N-15).

### Public database analysis

The following public databases were employed in this article: TIMER 2.0 (http://timer.cistrome.org/) 71, GEPIA (http://gepia.cancer-pku.cn/) 72 and R2: Genomics Analysis and Visualization Platform (http:// r2.amc.nl), which were employed to gather information on tumors from HNSCC patients.

### Interaction networks: BioGRID and IntAct

Interaction networks were constructed using the BioGRID and IntAct databases, along with the pandas packages to identify interactions with PSMG2. To visualize the networks, we employed the networkx and matplotlib packages in Python. Additionally, a Venn diagram was used to identify common protein interactions between the databases.

### GO and reactome enrichment analysis

Gene Ontology (GO) and Reactome Pathway enrichment analyses were performed using Gene Ontology (GO) and Reactome Pathway enrichment analyses were performed using the gseapy package. In this study, we selected the biological process (BP) category for GO analysis. A significance threshold of adjusted p-value < 0.05 was applied. Enrichment results were visualized using bar plots generated with the ggplot2 library in R, representing the 10 most significant pathways.

### Biocomputational analysis

To study the relationship between PSMG2 and the response to bortezomib, we analyzed the GSE9782 dataset. In this study, 188 patients received bortezomib as a treatment. The authors generated gene expression data during the course of American and international phases II and III clinical trials. The patients were classified based on relevant clinical variables, such as the days of tumor progression and the response to bortezomib. The MAS5.0-normalized data of the HG-U133A Affymetrix chip (GSE9782) were used in our study.

### Statistical analysis

Statistical analyses of experiments were performed using GraphPad Prism (6.01 for Windows). Control samples and tested samples were compared using unpaired Student's t test or Student's t test with Welch's correction, as appropriate. Experiments were performed a minimum of three times independently and in triplicate samples. P values less than 0.05 were considered statistically significant and were represented according to the following classification: p <0.05 (*), p <0.01 (**), and p <0.001 (***).

## Supplementary Material

Supplementary figures.

## Figures and Tables

**Figure 7:**
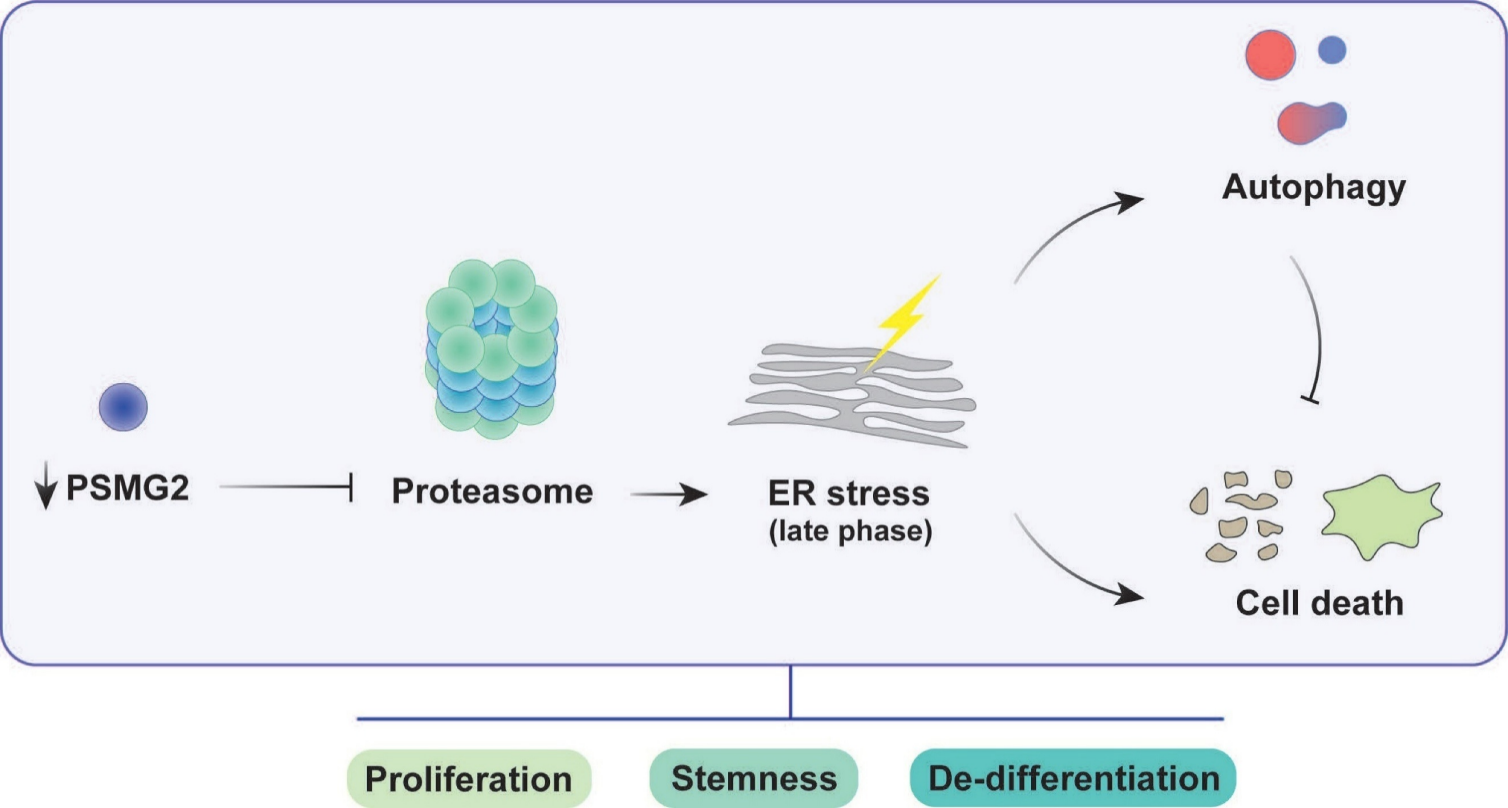
** Impact of PSMG2 on proteasome inhibition, ER stress, autophagy, and tumorigenesis in head and neck cancer cells.** The inhibition of proteasome activity caused by the reduction of PSMG2 levels activates ER stress, which in turn triggers increased autophagy and cell death. Autophagy functions as a protective mechanism by reducing protein load, maintaining cellular homeostasis, and promoting cell survival under stress conditions. However, the enhanced cell death, particularly through apoptosis and necrosis, results in a decrease in cell viability. This cascade of events leads to a reduction in cell proliferation both in vitro and in vivo. Furthermore, the diminished expression of pluripotency-associated genes and the loss of stemness properties contribute to the compromised tumorigenic potential of head and neck cancer cells. These combined effects suggest a critical role for PSMG2 in modulating cellular stress responses, autophagy, and stemness in cancer progression.

## Abbreviations

ALP: Alkaline Phosphatase
ATF4: Activating Transcription Factor 4
ATF6: Activating Transcription Factor 6
CHOP: C/EBP Homologous Protein
CRISPR: Clustered Regularly Interspaced Short Palindromic Repeats
CSCs: Cancer Stem cells
ER: Endoplasmic Reticulum
GRP78: Glucose regulated protein-78
HIF1a: Hypoxia inducible factor 1
HNSCC: Head and Neck Squamous Cell Carcinoma
HPV: Human papillomavirus
iPSc: Induced Pluripotent Stem Cells
LAMP-2: Lysosomal Associated Membrane Protein 2
LC3: Microtubule-associated protein 1A/1B-light chain 3 (LC3)
P62: Sequestosome 1 (SQSTM1)
PARP: Poly (ADP-ribose)
PERK: (Protein Kinase RNA-Like ER Kinase)
POMP: Proteasome Maturation Protein
ROS: Reactive Oxygen Species
PSMA5: Proteasome 20S Subunit Alpha 5
PSMA7/8: Proteasome 20S Subunit Alpha 7/8
PSMB1: Proteasome 20S Subunit Beta 1
PSMG1/2/3/4: Proteasome assembly chaperone 1/2/3/4
shRNA: Short hairpin RNA
UPR: Unfolded Protein Response
UPS: Ubiquitin Proteasome System

## References

[1] F (2012). Quail D, J. Taylor M, Postovit L-M. Microenvironmental Regulation of Cancer Stem Cell Phenotypes. Current Stem Cell Research & Therapy.

[2] Jopling C, Boue S, Belmonte JCI (2011). Dedifferentiation, transdifferentiation and reprogramming: three routes to regeneration. Nature Reviews Molecular Cell Biology.

[3] Hanahan D, Weinberg RA (2011). Hallmarks of cancer: the next generation. Cell.

[4] Hahn WC, Weinberg RA (2002). Rules for Making Human Tumor Cells. New England Journal of Medicine.

[5] Nowell PC (1976). The Clonal Evolution of Tumor Cell Populations. Science.

[6] Clarke MF, Dick JE, Dirks PB, Eaves CJ, Jamieson CHM, Jones DL (2006). Cancer Stem Cells-Perspectives on Current Status and Future Directions: AACR Workshop on Cancer Stem Cells. Cancer Res.

[7] Chaffer CL, Brueckmann I, Scheel C, Kaestli AJ, Wiggins PA, Rodrigues LO (2011). Normal and neoplastic nonstem cells can spontaneously convert to a stem-like state. Proc Natl Acad Sci U S A.

[8] Belgiovine C, Chiodi I, Mondello C (2008). Telomerase: cellular immortalization and neoplastic transformation. Multiple functions of a multifaceted complex. Cytogenetic and Genome Research.

[9] Sherr CJ, McCormick F (2002). The RB and p53 pathways in cancer. Cancer Cell.

[10] Carnero A, Hudson JD, Price CM, Beach DH (2000). p16INK4A and p19ARF act in overlapping pathways in cellular immortalization. Nature Cell Biology.

[11] García-Heredia JM, Verdugo Sivianes EM, Lucena-Cacace A, Molina-Pinelo S, Carnero A (2016). Numb-like (NumbL) downregulation increases tumorigenicity, cancer stem cell-like properties and resistance to chemotherapy. Oncotarget.

[12] Lleonart ME, Carnero A, Paciucci R, Wang Z-Q, Shomron N (2011). Cancer, senescence, and aging: translation from basic research to clinics. J Aging Res.

[13] Castro M, Martinez-Leal J, Lleonart M, Ramon Y Cajal S, Carnero A (2008). Loss-of-function genetic screening identifies a cluster of ribosomal proteins regulating p53 function. Carcinogenesis.

[14] Guijarro MV, Castro ME, Romero L, Moneo V, Carnero A (2007). Large scale genetic screen identifies MAP17 as protein bypassing TNF-induced growth arrest. Journal of Cellular Biochemistry.

[15] Chen W, Dong J, Haiech J, Kilhoffer M-C, Zeniou M (2016). Cancer Stem Cell Quiescence and Plasticity as Major Challenges in Cancer Therapy. Stem Cells Int.

[16] De Angelis ML, Francescangeli F, La Torre F, Zeuner A (2019). Stem Cell Plasticity and Dormancy in the Development of Cancer Therapy Resistance. Front Oncol.

[17] Sosa MS, Bragado P, Aguirre-Ghiso JA (2014). Mechanisms of disseminated cancer cell dormancy: an awakening field. Nat Rev Cancer.

[18] Sancho P, Barneda D, Heeschen C (2016). Hallmarks of cancer stem cell metabolism. Br J Cancer.

[19] Snyder V, Reed-Newman TC, Arnold L, Thomas SM, Anant S (2018). Cancer Stem Cell Metabolism and Potential Therapeutic Targets. Front Oncol.

[20] Peiris-Pagès M, Martinez-Outschoorn UE, Pestell RG, Sotgia F, Lisanti MP (2016). Cancer stem cell metabolism. Breast Cancer Research.

[21] Mody MD, Rocco JW, Yom SS, Haddad RI, Saba NF (2021). Head and neck cancer. The Lancet.

[22] Hammond EC, Horn D (1958). SMOKING AND DEATH RATES-REPORT ON FORTY-FOUR MONTHS OF FOLLOW-UP OF 187,783 MEN. Journal of the American Medical Association.

[23] Wyss A, Hashibe M, Chuang S-C, Lee Y-CA, Zhang Z-F, Yu G-P (2013). Cigarette, Cigar, and Pipe Smoking and the Risk of Head and Neck Cancers: Pooled Analysis in the International Head and Neck Cancer Epidemiology Consortium. American Journal of Epidemiology.

[24] Hashibe M, Brennan P, Benhamou S, Castellsague X, Chen C, Curado MP (2007). Alcohol Drinking in Never Users of Tobacco, Cigarette Smoking in Never Drinkers, and the Risk of Head and Neck Cancer: Pooled Analysis in the International Head and Neck Cancer Epidemiology Consortium. JNCI: Journal of the National Cancer Institute.

[25] Blot WJ, McLaughlin JK, Winn DM, Austin DF, Greenberg RS, Susan S (1988). Smoking and Drinking in Relation to Oral and Pharyngeal Cancer. Cancer Res.

[26] Chien YC, Chen JY, Liu MY, Yang HI, Hsu MM, Chen CJ (2001). Serologic markers of epstein-barr virus infection and nasopharyngeal carcinoma in taiwanese men. New England Journal of Medicine.

[27] Murata S, Yashiroda H, Tanaka K (2009). Molecular mechanisms of proteasome assembly. Nature Reviews Molecular Cell Biology.

[28] Hirano Y, Hendil KB, Yashiroda H, Iemura S-i, Nagane R, Hioki Y (2005). A heterodimeric complex that promotes the assembly of mammalian 20S proteasomes. Nature.

[29] Hirano Y, Hayashi H, Iemura S-i, Hendil KB, Niwa S-i, Kishimoto T (2006). Cooperation of Multiple Chaperones Required for the Assembly of Mammalian 20S Proteasomes. Molecular Cell.

[30] Le Tallec B, Barrault M-B, Courbeyrette R, Guérois R, Marsolier-Kergoat M-C, Peyroche A (2007). 20S Proteasome Assembly Is Orchestrated by Two Distinct Pairs of Chaperones in Yeast and in Mammals. Molecular Cell.

[31] Kusmierczyk AR, Kunjappu MJ, Funakoshi M, Hochstrasser M (2008). A multimeric assembly factor controls the formation of alternative 20S proteasomes. Nature structural & molecular biology.

[32] Yashiroda H, Mizushima T, Okamoto K, Kameyama T, Hayashi H, Kishimoto T (2008). Crystal structure of a chaperone complex that contributes to the assembly of yeast 20S proteasomes. Nature structural & molecular biology.

[33] Hirano Y, Kaneko T, Okamoto K, Bai M, Yashiroda H, Furuyama K (2008). Dissecting beta-ring assembly pathway of the mammalian 20S proteasome. EMBO J.

[34] Li X, Kusmierczyk AR, Wong P, Emili A, Hochstrasser M (2007). beta-Subunit appendages promote 20S proteasome assembly by overcoming an Ump1-dependent checkpoint. EMBO J.

[35] Li X, Li Y, Arendt CS, Hochstrasser M (2016). Distinct Elements in the Proteasomal β5 Subunit Propeptide Required for Autocatalytic Processing and Proteasome Assembly. J Biol Chem.

[36] Ramos PC, Höckendorff J, Johnson ES, Varshavsky A, Dohmen RJ (1998). Ump1p Is Required for Proper Maturation of the 20S Proteasome and Becomes Its Substrate upon Completion of the Assembly. Cell.

[37] Yang Y, Früh K, Ahn K, Peterson PA (1995). In Vivo Assembly of the Proteasomal Complexes, Implications for Antigen Processing (*). Journal of Biological Chemistry.

[38] Smith MH, Ploegh HL, Weissman JS (2011). Road to Ruin: Targeting Proteins for Degradation in the Endoplasmic Reticulum. Science.

[39] Oslowski CM, Urano F (2011). Chapter Four - Measuring ER Stress and the Unfolded Protein Response Using Mammalian Tissue Culture System. In: Conn PM, editor. Methods in Enzymology: Academic Press.

[40] Shen J, Snapp EL, Lippincott-Schwartz J, Prywes R (2005). Stable Binding of ATF6 to BiP in the Endoplasmic Reticulum Stress Response. Molecular and Cellular Biology.

[41] Harding HP, Zhang Y, Bertolotti A, Zeng H, Ron D (2000). <em>Perk</em> Is Essential for Translational Regulation and Cell Survival during the Unfolded Protein Response. Molecular Cell.

[42] Shin WH, Park JH, Chung KC (2020). The central regulator p62 between ubiquitin proteasome system and autophagy and its role in the mitophagy and Parkinson's disease. BMB Rep.

[43] Wang DW, Peng ZJ, Ren GF, Wang GX (2015). The different roles of selective autophagic protein degradation in mammalian cells. Oncotarget.

[44] Lim J, Lachenmayer ML, Wu S, Liu W, Kundu M, Wang R (2015). Proteotoxic stress induces phosphorylation of p62/SQSTM1 by ULK1 to regulate selective autophagic clearance of protein aggregates. PLoS Genet.

[45] Moscat J, Diaz-Meco MT, Albert A, Campuzano S (2006). Cell Signaling and Function Organized by PB1 Domain Interactions. Molecular Cell.

[46] Assou S, Cerecedo D, Tondeur S, Pantesco V, Hovatta O, Klein B (2009). A gene expression signature shared by human mature oocytes and embryonic stem cells. BMC Genomics.

[47] Babaie Y, Herwig R (2007). Analysis of Oct4-dependent transcriptional networks regulating self-renewal and pluripotency in human embryonic stem cells. Stem Cells.

[48] Baharvand H, Hajheidari M, Ashtiani SK, Salekdeh GH (2006). Proteomic signature of human embryonic stem cells. PROTEOMICS.

[49] Szutorisz H, Georgiou A, Tora L, Dillon N (2006). The Proteasome Restricts Permissive Transcription at Tissue-Specific Gene Loci in Embryonic Stem Cells. Cell.

[50] Buckley Shannon M, Aranda-Orgilles B, Strikoudis A, Apostolou E, Loizou E, Moran-Crusio K (2012). Regulation of Pluripotency and Cellular Reprogramming by the Ubiquitin-Proteasome System. Cell Stem Cell.

[51] Barrandon Y, Green H (1987). Three clonal types of keratinocyte with different capacities for multiplication. Proceedings of the National Academy of Sciences.

[52] Locke M, Heywood M, Fawell S, Mackenzie IC (2005). Retention of Intrinsic Stem Cell Hierarchies in Carcinoma-Derived Cell Lines. Cancer Res.

[53] Beaver CM, Ahmed A, Masters JR (2014). Clonogenicity: Holoclones and Meroclones Contain Stem Cells. PLOS ONE.

[54] Navas LE, Blanco-Alcaina E, Suarez-Martinez E, Verdugo-Sivianes EM, Espinosa-Sanchez A, Sanchez-Diaz L (2023). NAD pool as an antitumor target against cancer stem cells in head and neck cancer. J Exp Clin Cancer Res.

[55] Sasaki K, Hamazaki J, Koike M, Hirano Y, Komatsu M, Uchiyama Y (2010). PAC1 Gene Knockout Reveals an Essential Role of Chaperone-Mediated 20S Proteasome Biogenesis and Latent 20S Proteasomes in Cellular Homeostasis. Molecular and Cellular Biology.

[56] Fribley A, Wang C-Y (2006). Proteasome inhibitor induces apoptosis through induction of endoplasmic reticulum stress. Cancer Biology & Therapy.

[57] Walter P, Ron D (2011). The Unfolded Protein Response: From Stress Pathway to Homeostatic Regulation. Science.

[58] Cohen-Kaplan V, Ciechanover A, Livneh I (2017). Stress-induced polyubiquitination of proteasomal ubiquitin receptors targets the proteolytic complex for autophagic degradation. Autophagy.

[59] Fukusumi T, Ishii H, Konno M, Yasui T, Nakahara S, Takenaka Y (2014). CD10 as a novel marker of therapeutic resistance and cancer stem cells in head and neck squamous cell carcinoma. Br J Cancer.

[60] Piattelli A, Fioroni M, Iezzi G, Perrotti V, Stellini E, Piattelli M (2006). CD10 expression in stromal cells of oral cavity squamous cell carcinoma: a clinic and pathologic correlation. Oral Diseases.

[61] Faber A, Aderhold C, Goessler UR, Hoermann K, Schultz JD, Umbreit C (2014). Interaction of a CD44+ head and neck squamous cell carcinoma cell line with a stromal cell-derived factor-1-expressing supportive niche: An in vitro model. Oncol Lett.

[62] Matsumoto K, Nishiya T, Maekawa S, Horinouchi T, Ogasawara K, Uehara T (2011). The ECS(SPSB) E3 ubiquitin ligase is the master regulator of the lifetime of inducible nitric-oxide synthase. Biochemical and Biophysical Research Communications.

[63] Matsuoka S, Oike Y, Onoyama I, Iwama A, Arai F, Takubo K (2008). Fbxw7 acts as a critical fail-safe against premature loss of hematopoietic stem cells and development of T-ALL. Genes Dev.

[64] Crusio KM, King B, Reavie LB, Aifantis I (2010). The ubiquitous nature of cancer: the role of the SCFFbw7 complex in development and transformation. Oncogene.

[65] Thompson BJ, Jankovic V, Gao J, Buonamici S, Vest A, Lee JM (2008). Control of hematopoietic stem cell quiescence by the E3 ubiquitin ligase Fbw7. J Exp Med.

[66] Kimura Y, Tanaka K (2010). Regulatory mechanisms involved in the control of ubiquitin homeostasis. The Journal of Biochemistry.

[67] Vilchez D, Boyer L, Morantte I, Lutz M, Merkwirth C, Joyce D (2012). Increased proteasome activity in human embryonic stem cells is regulated by PSMD11. Nature.

[68] Atkinson SP, Collin J, Irina N, Anyfantis G, Kyung BK, Lako M (2012). A Putative Role for the Immunoproteasome in the Maintenance of Pluripotency in Human Embryonic Stem Cells. STEM CELLS.

[69] Ruiz L (2008). Characterization of the p53 response to oncogene-induced senescence. PLoS One.

[70] Khan AA, Dwivedi S, Singh S, Kumar M, Trivedi SP (2025). Perturbations in Redox Status, Biochemical Indices, and Expression of XBP1s and NOX4 in the Liver of Channa Punctatus Following Exposure to Mancozeb. Gene Expression.

[71] Li T, Fan J, Wang B, Traugh N, Chen Q, Liu JS (2017). TIMER: A Web Server for Comprehensive Analysis of Tumor-Infiltrating Immune Cells. Cancer Res.

[72] Tang Z, Li C, Kang B, Gao G, Li C, Zhang Z (2017). GEPIA: a web server for cancer and normal gene expression profiling and interactive analyses. Nucleic Acids Research.

