# Supplementary material for: PSMG2 role in tumorigenesis and stemness mediated by protein accumulation, reticulum stress and autophagy
Source: Int J Biol Sci. 2025 Mar 21;21(6):2531–49. doi: 10.7150/ijbs.105263 (PMC12035902; doi:10.7150/ijbs.105263)
Supplement: Supplementary file 1 — Supplementary figures. [file ijbsv21p2531s1.pdf]

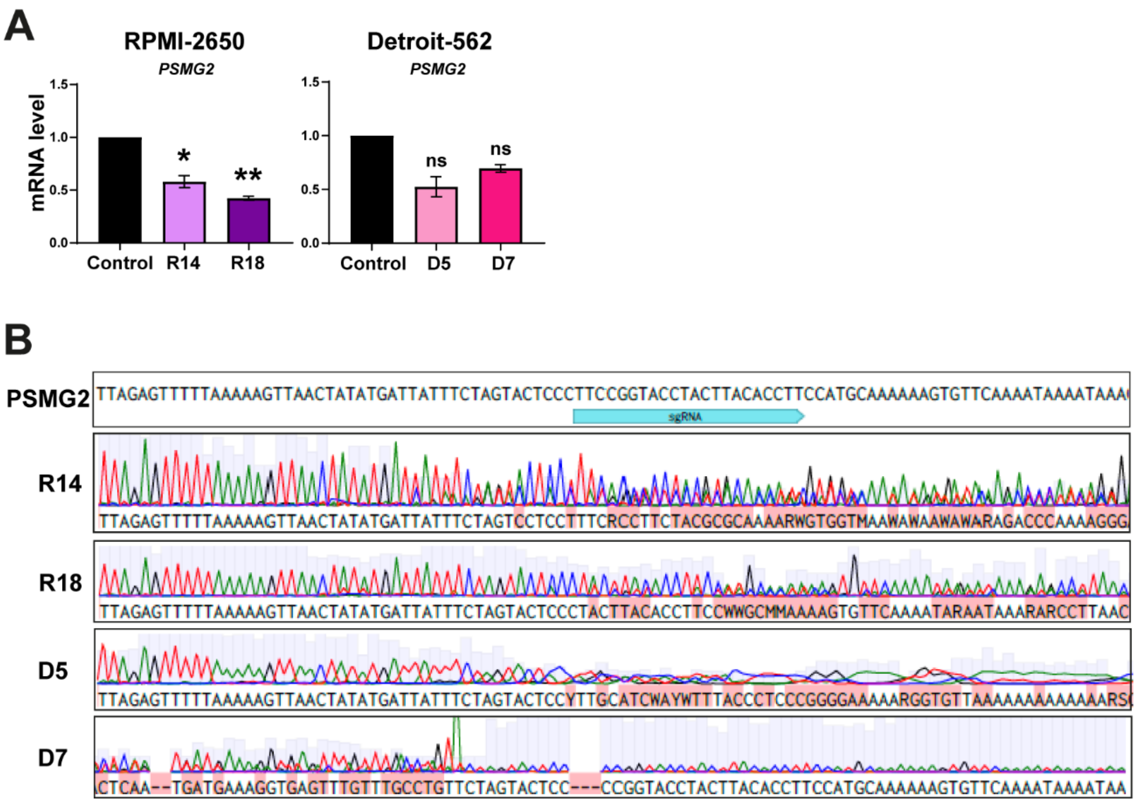

Figure S1. (A) Validation of the reduction in PSMG2 expression in CRISPR PSMG2 cells of the RPMI-2650 and Detroit-562 head-neck cancer cell lines by RT–PCR. **(B)** Part of DNA sequence which containing the sgRNA sequence (indicated in blue) in original sequence of PSMG2 to compare with the sequence of R14, R18, D5 and D7 clones. Behind the sgRNA sequence, it is found nucleotide sequence modified by cas9 cut site (indicated in red). The mean of a minimum of 3 independent experiments performed in triplicate ± standard error is represented in all experiments. Statistical analysis was performed with the t-Student test, ns: non-significant, * $p < 0.05$, ** $p < 0.01$.

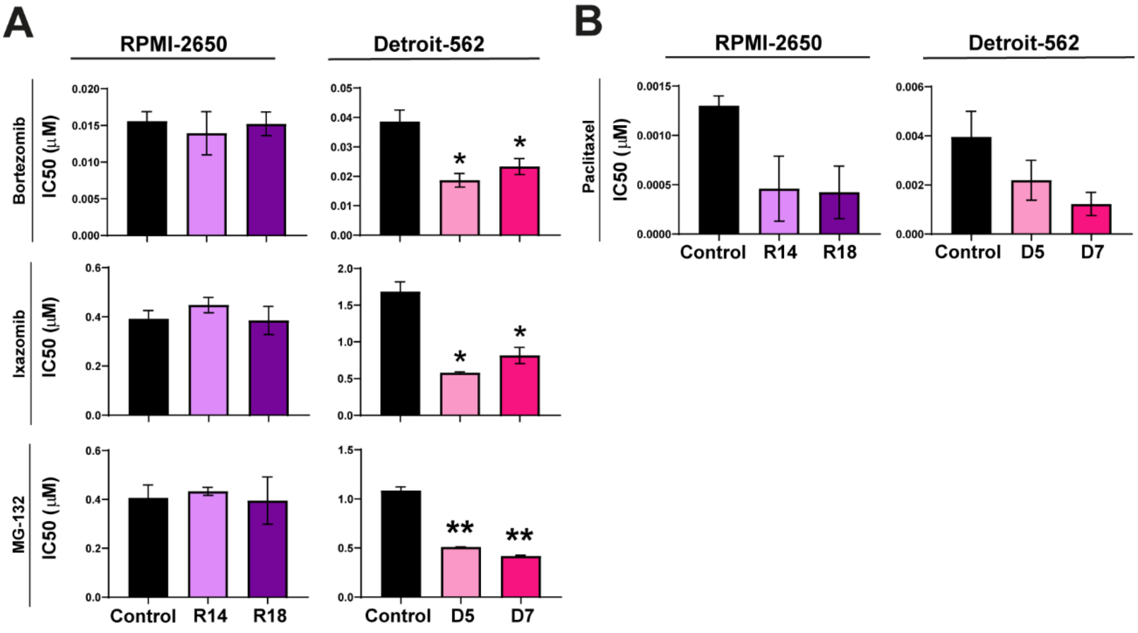

Figure S2. Impact of PSMG2 dowregulation on chemotherapeutic drug response. (A-C) IC50 of proteasome inhibitors **(A)** and paclitaxel **(B)** in control and PSMG2 CRISPRs. The mean of a minimum of 3 independent experiments performed in triplicate ± standard error is shown in all experiments. Statistical analysis was performed with Student's t test, ns: non-significant, * $p < 0.05$.

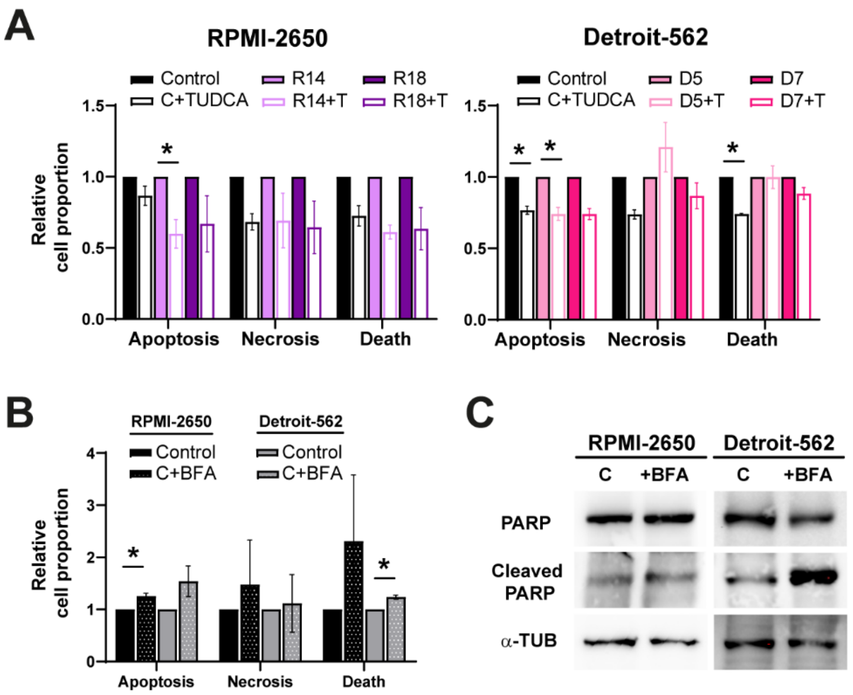

Figure S3. Analysis of cell death induced by ER stress modulation using TUDCA and BFA. (A) Proportion of cell death in RPMI-2650 and Detroit-562 control cells, CRISPRs PSMG2 and control and CRISPRs cells treated with TUDCA (+T) by FACS. **(B)** Proportion of cell death in RPMI-2650 and Detroit-562 control cells and cells treated with BFA by FACS. **(C)** Western blot analysis of PARP in control and BFA-treated cells. C/Control (parental cell line), +T (cell line treated with TUDCA), +BFA (cell line treated with BFA). The mean of a minimum of 3 independent experiments performed in triplicate ± standard error is represented in all experiments. Statistical analysis was performed with Student's t test, * $p < 0.05$, ** $p < 0.01$.

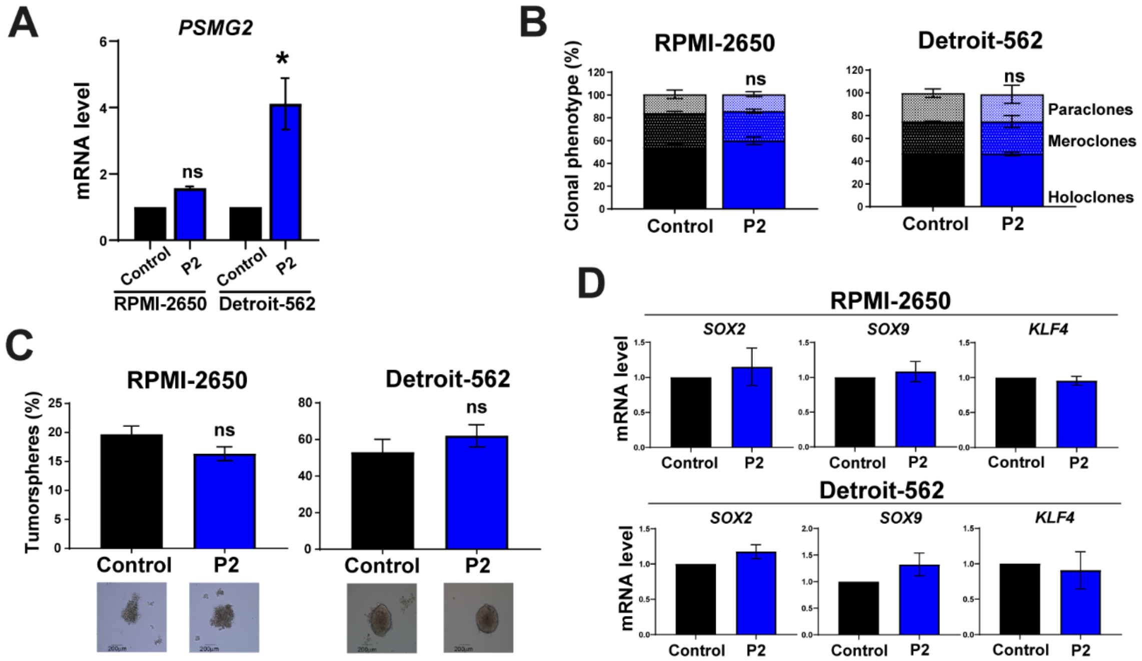

Figure S4 (A) Validation of the overexpression of PSMG2 in cells of the RPMI-2650 and Detroit-562 head-neck cancer cell lines by RT–PCR**. (B)** Percentages of holoclones, meroclones and paraclones generated by RPMI-2650 and Detroit-562 control and overexpressed PSMG2 cell lines seeded at low density over 15 days. **(C)** Percentages of tumorspheres formed from the whole population of RPMI-2650 and Detroit-562

control and overexpressed PSMG2 cell lines. Representative images of the tumorspheres are shown (scale bars: 200 µm). **(D)** Measurement of *SOX2*, *SOX9*, *KLF4* and *NANOG* expression levels by RT-qPCR in RPMI-2650 and Detroit-562 control and overexpressed PSMG2 cell lines. Graphs represent mRNA levels in overexpression of PSMG2 cells normalized to the mRNA levels of control cells. The mean of a minimum of 3 independent experiments performed in triplicate ± standard error is represented in all experiments. Statistical analysis was performed with the t-Student test, ns: non-significant.

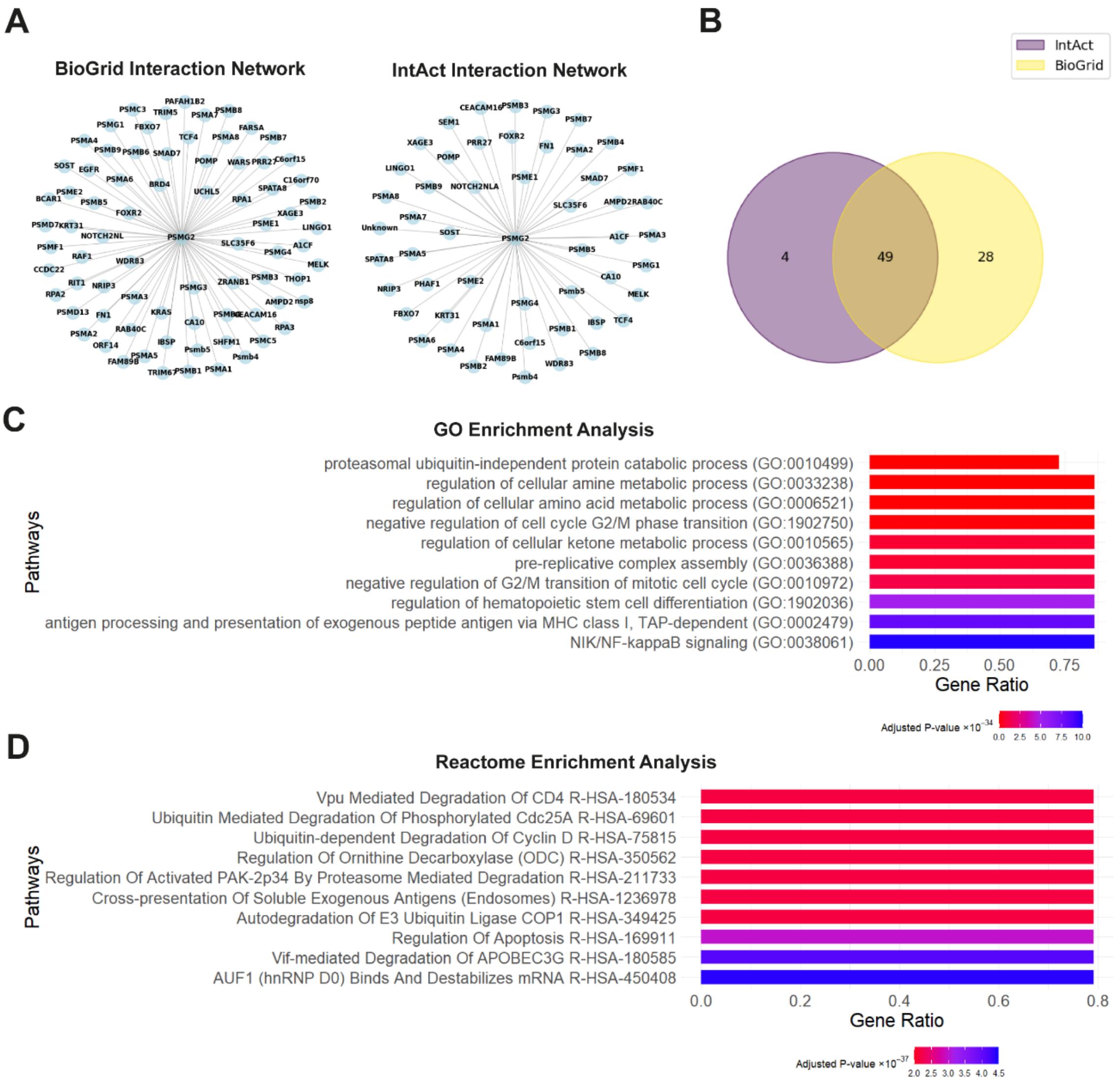

Figure S5. Integration of PSMG2 interactome data from BioGRID and IntAct databases. (A) Network interaction graphs were generated for each database to illustrate the connectivity and relationships among the interacting proteins. **(B)** A Venn diagram was constructed to visualize the overlap of interactions identified in both databases. **(C-D)** Genes that were common to both datasets were selected for Gene Ontology (GO) enrichment analysis **(C)** and Reactome pathway analysis **(D)**.

Acronyms

**ACC**	Adrenocortical Carcinoma
**BLCA**	Bladder Urothelial Carcinoma
**BRCA**	Breast Invasive Carcinoma
**CESC**	Cervical and Endocervical Cancer
**CHOL**	Cholangiocarcinoma
**COAD**	Colon Adenocarcinoma
**DLBC**	Diffuse Large B-cell Lymphoma
**ESCA**	Esophageal Carcinoma
**GBM**	Glioblastoma Multiforme
**HNSCC**	Head and Neck Squamous Cell Carcinoma
**KICH**	Kidney Chromophobe
**KIRC**	Kidney Renal Clear Cell Carcinoma
**KIRP**	Kidney Renal Papillary Cell Carcinoma
**LAML**	Acute Myeloid Leukemia
**LGG**	Lower Grade Glioma
**LIHC**	Liver Hepatocellular Carcinoma
**LUAD**	Lung Adenocarcinoma
**LUSC**	Lung Squamous Cell Carcinoma
**MESO**	Mesothelioma
**OV**	Ovarian Serous Cystadenocarcinoma
**PAAD**	Pancreatic Adenocarcinoma
**PCPG**	Pheochromocytoma and Paraganglioma
**PRAD**	Prostate Adenocarcinoma
**READ**	Rectum Adenocarcinoma
**SARC**	Sarcoma
**SKCM**	Skin Cutaneous Melanoma
**STAD**	Stomach Adenocarcinoma
**TGCT**	Testicular Germ Cell Tumors
**THCA**	Thyuoid Carcinoma
**THYM**	Thymoma
**UCEC**	Uterine Corpus Endometrial Carcinoma
**UCS**	Uterine Carsinosarcoma
**UVM**	Uveal Melanoma
